# Integrating natural non-pharmaceutical therapies into medical tourism: a dynamic health portrait-driven model for proactive older adult health and public health services

**DOI:** 10.3389/fpubh.2025.1580082

**Published:** 2025-10-09

**Authors:** Mingyu Liu, Boyuan Wang

**Affiliations:** ^1^College of International Tourism and Public Administration, Hainan University, Haikou, China; ^2^Beijing Xiaotangshan Hospital, Beijing, China; ^3^Department of Biomedical Sciences, Jockey Club College of Veterinary Medicine and Life Sciences, City University of Hong Kong, Hong Kong, China

**Keywords:** medical tourism, non-pharmacological therapies, older adult patients with chronic diseases, public health, cost-effectiveness

## Abstract

In the face of the dual challenges of global population aging and the high prevalence of chronic diseases, traditional healthcare systems are facing severe tests in terms of resource allocation and service accessibility. This study focuses on the synergistic integration of natural non-pharmacological therapies (NPT) and medical tourism (MT), exploring their potential as an innovative service model to enhance the proactive health management capabilities of older adult patients with chronic conditions and optimize public health management. MT integrates global high-quality medical resources with personalized services (such as traditional Chinese medicine, physical therapy, psychological therapy, hydrotherapy, nutritional management, and other diversified NPT) to provide older populations with comprehensive health interventions encompassing sports activities and rehabilitation, nutritional dietary management, social interaction and mental health, emotional adjustment, and physical therapy. A systematic review of the literature revealed that this model has achieved significant results in promoting early intervention for chronic diseases, improving the physical and mental health of the people over 65, and enhancing social functioning and quality of life. However, it still has limitations in terms of standardized construction (especially the integration of traditional Chinese medicine), the depth of interdisciplinary collaboration, evidence-based research on long-term health benefits, and the development of intelligent and precise services. Future development should focus on establishing a multidisciplinary NPT-MT service standard system, strengthening interdisciplinary evidence-based research (especially long-term effect assessments), and exploring intelligent algorithm-driven dynamic health management pathways. This study provides theoretical basis and practical references for policymakers to optimize healthy aging strategies, healthcare institutions to innovate service models, and related industries to develop integrated health tourism products.

## 1 Introduction

Despite significant advancements in modern medical technology, the current healthcare system still faces numerous challenges in chronic disease management, resource allocation, and service accessibility (1, 2). First, the traditional disease-centered model places insufficient emphasis on preventive health management, contributing to high chronic disease prevalence and long-term patient reliance on medication or costly treatments (3–5). This “symptom-focused” approach overlooks the importance of early intervention and continuous monitoring in reducing chronic disease risks. Second, global disparities in healthcare resource distribution are pronounced. High medical costs, scarcity of quality resources, and their concentration in urban areas create common challenges for many countries (5–7). These issues are exacerbated by the growing trend of population aging, which further widens the imbalance between increasing healthcare demand and limited supply. Additionally, long waiting times, overburdened healthcare facilities, and a lack of focus on personalized health management have driven many patients to seek more flexible and efficient solutions (7, 8). This highlights the limitations of the existing system in addressing diverse health needs and underscores the public’s urgent desire for improved service quality. While medical technology and public health systems have made considerable progress, the dual challenges of aging populations and rising chronic disease rates persist (9). To address these issues effectively, innovative strategies and policy support are needed to shift from treatment-focused care toward comprehensive prevention and personalized health management.

According to the latest estimates by the World Health Organization (10), the global population aged 60 and above reached 1 billion in 2019 and is projected to grow to 2.1 billion by 2050. This significant aging trend poses profound challenges to public health and socio-economic systems. Specifically, aging has accelerated the prevalence of chronic diseases and associated mortality rates, with dementia standing out as a particularly critical issue. Currently, approximately 50 million people worldwide are affected by dementia, and in China, the dementia prevalence rate among those aged 60 and above has reached 6.0% (11). It is estimated that by 2030, the annual economic cost related to dementia will rise to $114.2 billion. Moreover, population aging exerts multifaceted pressures on labor markets, economic growth, and government finances (12). Research indicates that unhealthy lifestyles, such as physical inactivity and imbalanced diets (9), further exacerbate the risk of chronic diseases, thereby intensifying the health burden in aging societies.

In response, healthcare paradigms must evolve beyond episodic treatment into continuous, proactive health management systems that accommodate the dynamic and personalized needs of aging individuals. In this context, Artificial Intelligence (AI) and continual learning models offer transformative potential, particularly when integrated with personalized health services like medical tourism (MT) and natural non-pharmaceutical therapies. Continual learning, defined as the ability of AI systems to progressively learn from evolving data streams across multiple tasks, addresses key real-world challenges such as concept drift, data heterogeneity, and long-term adaptability. It is especially relevant for older adults health management, where health status fluctuates over time, and conventional static models fall short in responsiveness and accuracy.

To bridge this gap, we propose an AI-empowered framework for proactive older adult health management, featuring a dynamic health portrait model based on four dimensions of data integration—environmental exposure, behavioral patterns, physiological signals, and feedback loops. This model leverages continual learning algorithms to enable real-time monitoring, cross-context adaptation, and personalized intervention across medical tourism scenarios. Such a system can adapt to patient needs across time and space, offering a scalable, intelligent foundation for next-generation public health services.

Chronic disease management is a critical component of public health administration and a key approach to slowing the progression of major illnesses (13). Natural therapy, as an intervention method based on natural factors, has demonstrated significant efficacy in managing chronic diseases, improving physical and mental well-being, and preventing illness. This therapeutic approach utilizes natural elements closely related to human life, such as food, air, water, and sunlight (14), combined with exercises, sleep, rest, and positive psychological factors to promote health. Specifically, natural therapy encompasses various forms, including oxygen therapy, light therapy, hydrotherapy, and thermotherapy. Modern natural therapy often integrates herbal medicine, dietary nutrition, lifestyle modifications, and personalized treatments such as massage and yoga (14). The core philosophy of natural therapy is grounded in six principles: the healing power of nature (vis medicatrix naturae), identifying and treating the root cause (tolle causam), the primary principle of “do no harm” (primum non nocere), the role of the physician as an educator (docere), holistic treatment (treat the whole person), and disease prevention (15). These principles emphasize encouraging the body’s innate self-healing mechanisms and employing non-invasive, low-risk methods to achieve disease prevention, treatment, and optimal health promotion (16).

Existing research demonstrates that natural therapy holds significant advantages across various health domains. For instance, Hussam (17) highlight that compared to conventional medication, natural therapy offers greater accessibility and lower costs, making it an increasingly popular intervention for managing chronic conditions such as hypertension. Additionally, many researchers found that exposure to natural landscapes positively impacts mental health by promoting perceived recovery (18–20). This finding aligns with the Attention Restoration Theory (ART) (21) and Stress Recovery Theory (SRT) (22), which suggest that contact with nature-rich environments effectively alleviates psychological stress, improves emotional states, and enhances cognitive function. In pharmacological treatments, numerous studies have confirmed the efficacy of certain plant-based compounds in disease management and anti-aging interventions. For example, extracts from plants such as ginseng, *Catha edulis*, and *Nigella sativa* have been shown to exhibit therapeutic effects on diabetes, chronic diseases, and even cancer (23–25). Furthermore, natural therapy has been widely adopted as an adjunct treatment for neurodegenerative diseases (26, 27), providing patients with diverse treatment options. Notably, MT, as an emerging health management model, integrates the aforementioned natural therapies, offering innovative solutions for global chronic disease management and health promotion. This approach not only enhances the accessibility of natural therapies but also fosters cross-cultural exchanges in healthcare services, contributing to the development of more inclusive and effective global health strategies.

MT and health tourism have emerged as novel forms of natural therapy, experiencing sustained growth in demand in recent years (28, 29). Health tourism is defined as an organized form of travel aimed at maintaining, enhancing, or restoring an individual’s physical and mental well-being through a change in environment (30). This concept encompasses MT (31) and emphasizes the promotion of overall health through exposure to natural environments and lifestyle interventions. In contrast, MT specifically refers to domestic or international travel for the purpose of obtaining medical services, with core components including surgery, treatment, rehabilitation, and health management (8, 32, 33). From these definitions, it is evident that health tourism has a broader scope, encompassing multiple dimensions of healthcare, while MT primarily focuses on disease treatment and recovery (34).

Thus, to enhance the health and Quality of Life (QoL) for older adults, the healthcare system urgently needs to transition from a traditional, single-disease treatment model to an integrated health management approach. In this context, MT, as a multidimensional model that combines medical care, rehabilitation, and health promotion, offers a viable solution to address the health challenges posed by aging societies. Compared to conventional healthcare systems, MT presents significant advantages, including cost-effectiveness, advanced technology applications, shorter waiting times, and comprehensive rehabilitation services (35–39). These features enable patients to access high-quality medical care while achieving holistic improvements in physical and mental well-being. Particularly for patient groups requiring surgical interventions, chronic disease management, or alternative therapies, health tourism not only provides more diverse options but also effectively alleviates the strain on domestic medical resources (32, 33). By integrating medical needs with travel experiences, health tourism satisfies personalized healthcare demands and offers a new perspective for optimizing the global allocation of medical resources.

**Figure 1:**
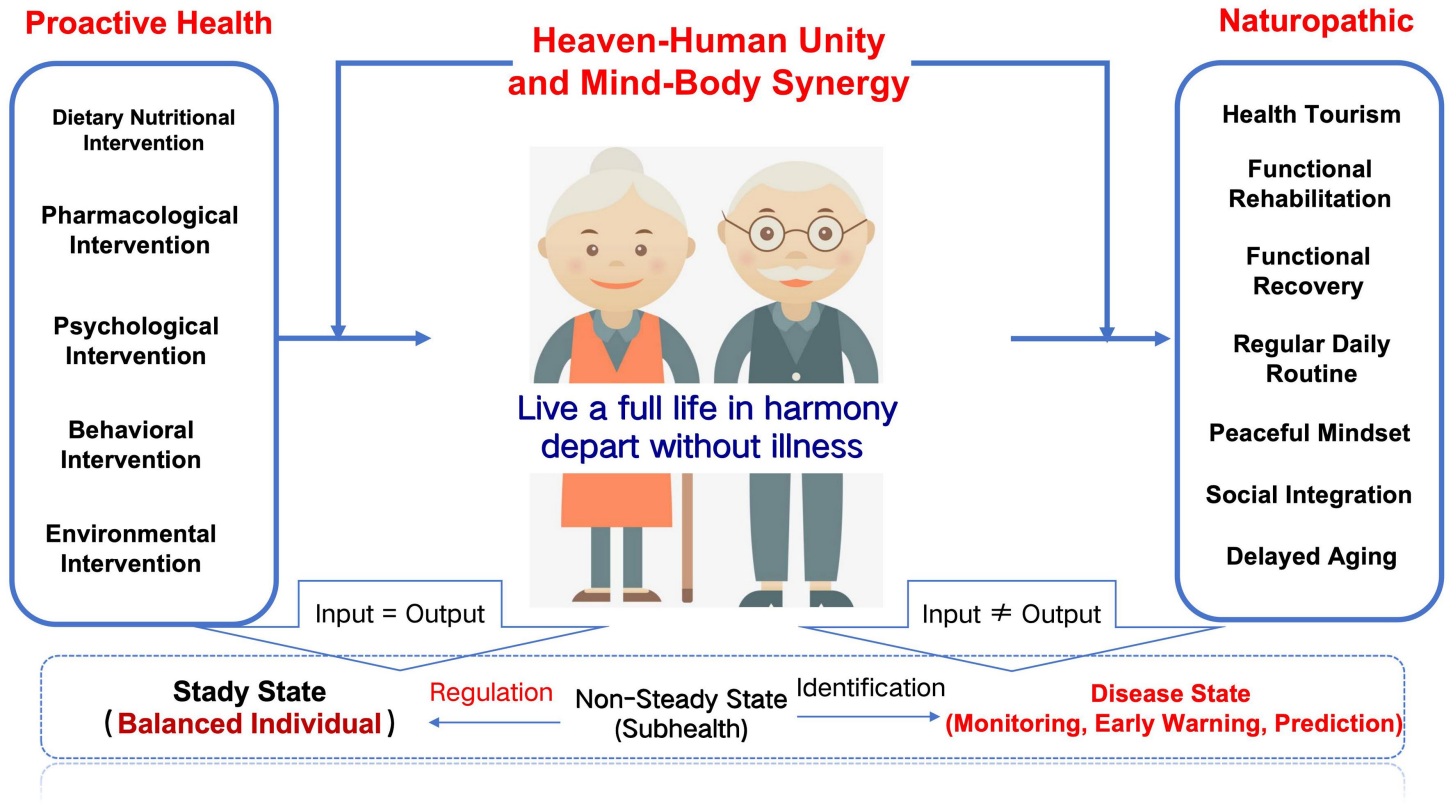
Schematic diagram of active health for older population with chronic diseases. For older adult patients with chronic diseases, an integrated model combining non-pharmacological and pharmacological therapies for proactive health management is essential. To address the issue of multimorbidity in older adults, it is critical to prioritize the enhancement of intrinsic capacity as a foundation. This involves developing a new model of collaborative intervention that integrates non-pharmacological and pharmacological therapies. The goal is to detect potential health risks early, promptly address hidden health issues (“invisible bombs”), improve QoL, reduce years lived with disability (YLD), and increase healthy life expectancy. By adopting this comprehensive approach, older individuals can achieve better overall health outcomes while managing their chronic conditions effectively.

The improvement of health in older populations with chronic diseases cannot rely solely on traditional passive medical models but must shift toward proactive health management. Proactive health represents a responsibility-driven management approach centered on the individual, emphasizing the adoption of healthy lifestyles and behaviors combined with modern medical interventions to achieve disease prevention and treatment goals (40). As illustrated in Figure 1, this concept is particularly crucial in the context of the increasing prevalence of chronic diseases among younger populations. It challenges the conventional notion of seeking medical care only when illness occurs and aligns closely with the preventive philosophy of Traditional Chinese Medicine, specifically the concept of “treating pre-disease” (41). Within this framework, MT emerges as an innovative health management model that plays a significant role in promoting proactive health management in aging societies. By integrating global high-quality medical resources with personalized services, MT not only offers patients more comprehensive treatment options but also drives innovation and development within global healthcare systems (42–47). This approach shifts health management from reactive treatment to proactive prevention, providing new strategies to address the health challenges posed by population aging.

This study aims to explore how health and MT, grounded in the proactive health theory and incorporating integrative East–West medicine-based natural therapies, can effectively address public health management challenges faced by aging societies in the context of global population aging and the growing health industry. By systematically reviewing relevant literature on natural therapies and MT, this research analyzes their applications in chronic disease management, health promotion, and healthy aging. It provides theoretical support for understanding the role of MT in enhancing the QoL for older adults, promoting active aging, and driving innovation in health management systems.

The contributions of this study are reflected in the following aspects:

1. Through systematic study of peer-reviewed literature in the field of MT, we have innovatively refined a four-dimensional data fusion framework of “environment-behavior-physiology-feedback”; based on this framework, we have constructed for the first time a generic model of dynamic health profiling for MT that can be reused across scenarios. Based on this framework, we constructed the first generic model of dynamic health portrait that can be reused across scenarios. Through standardized data-driven and personalized intervention mechanisms, this model not only promotes the transformation of medical tourism from static experience to dynamic management, but also introduces an AI-driven evolvable mechanism for the public health service system.
2. This study analyzes the synergistic efficacy of integrating natural non-pharmacological therapies in the medical tourism ecosystem, including the principles of Traditional Chinese Medicine (TCM), complementary methods of Western medicine, and proactive health interventions. The study demonstrated the optimization of medical tourism public services, uniquely revealing the synergistic potential of non-pharmacological therapies in improving key geriatric health indicators, such as functional maintenance, psychological balance, social integration, and delayed aging, as well as significantly enhancing the efficacy and personalization of medical tourism public services, in line with traditional Chinese medicine’s principles of “unity of man and nature” and “mind and body.” This is in line with the traditional Chinese medicine principles of “unity of heaven and mankind” and “synergy of mind and body.
3. This study identifies gaps in the current research at the intersection of MT and healthy aging, proposing future research directions. The findings offer valuable theoretical support and practical references for policymakers, healthcare institutions, and related industries, promoting innovation and sustainable development in MT models. Through this, the study contributes to advancing both academic understanding and real-world applications in the field.

Based on the clarification of the research background, core issues, and significance of this study, the overall research framework and core content of this paper are outlined below to clearly present the research context and guide subsequent analysis: Chapter 2 systematically establishes the theoretical foundation of the study. Chapter 3 details the systematic literature review methodology followed in this study, which adheres to the PRISMA framework. Chapter 4 is the core empirical analysis section of the study. Chapter 5 Based on the preceding analysis, this chapter objectively examines the primary limitations of the current synergistic application of MT and NPT in the field of proactive health management for older adult patients with chronic conditions and proposes forward-looking future research directions accordingly. Chapter 6 summarizes the entire paper, distilling the main findings, theoretical contributions, and practical implications of the research.

## 2 Fundamental theory

This chapter aims to systematically elaborate on the core concepts and theoretical foundations of this study, providing a theoretical basis for subsequent analyses of the synergistic effects of natural non-pharmacological therapies and MT in the active health management of older adult patients with chronic conditions and public health. Given that this study focuses on health promotion for the older adults with chronic conditions, this chapter will first define the key vehicles and core intervention methods of the study, then delve into the core health objectives (healthy aging) of the target population (older adults with chronic health conditions), and finally clarify the key pathways (active health management) to achieve these objectives. Specifically, the content of this chapter is organized as follows: Section 1 (2.1) defines the conceptual frameworks, development trends, and integrated applications of MT and non-pharmacological therapies in modern health management, laying the foundation for the service formats and intervention methods of this study; Section 2 (2.2) analyzes the scientific implications, challenges, and implementation pathways of healthy aging, particularly the core role of non-pharmacological interventions in extending healthy life expectancy, enhancing functional capacity, and improving quality of life, clarifying the ultimate goal orientation of the study; Section 3 (2.3) explains the concept, model, and value of active health management in chronic disease management and public health, emphasizing the importance of individual active participation and social support systems, and provides a framework for understanding how the synergistic effects of MT and NPT can be embedded in and promote active health management at both the individual and group levels; Section 4 (2.4) establishes the theoretical basis of continual learning, emphasizing its role in enabling adaptive, self-evolving health models that can dynamically incorporate multimodal data from natural non-pharmacological interventions while mitigating catastrophic forgetting and concept drift. These four sections build upon one another, addressing the core research questions from three dimensions: “tools and methods” (MT/natural non-pharmacological therapies), “goals and challenges” (healthy aging), and “pathways and frameworks” (active health management), collectively forming the theoretical framework supporting the core research questions.

### 2.1 MT and NPT

Despite the growing global trend of population aging and increasing attention to physical and mental well-being, MT has emerged as a significant research focus. However, the lack of a unified and standardized definition of MT in academia presents numerous challenges for both research and destination development (33, 48–50). In this study, MT is defined as domestic or international travel undertaken by individuals to access medical services, encompassing specific interventions such as surgery, treatment, rehabilitation, and health management (8, 32, 33). The growth of MT relies on several key factors, including access to high-quality yet affordable medical resources, shorter waiting times, and integrated medical and tourism service models (7, 8, 50, 51). With the acceleration of globalization, intensifying market competition, and rapid advancements in transportation, communication, and information technology, cross-border healthcare services have experienced substantial growth (52), further driving the expansion and prosperity of the MT industry.

In recent years, MT has expanded beyond traditional healthcare services to incorporate NPT, such as TCM, physiotherapy, psychotherapy, balneotherapy, horticultural therapy, and dietary therapy (31, 36, 47, 50, 53–61). These therapeutic approaches not only diversify the service offerings within MT but also provide medical tourists with more comprehensive and personalized health management solutions, better addressing their varied needs. By integrating NPT into its framework, MT enhances its capacity to promote holistic well-being, making it an increasingly attractive option for individuals seeking alternative or complementary healthcare interventions. This evolution reflects a broader shift toward proactive health management, emphasizing prevention, rehabilitation, and overall wellness in addition to conventional treatment modalities.

**Figure 2:**
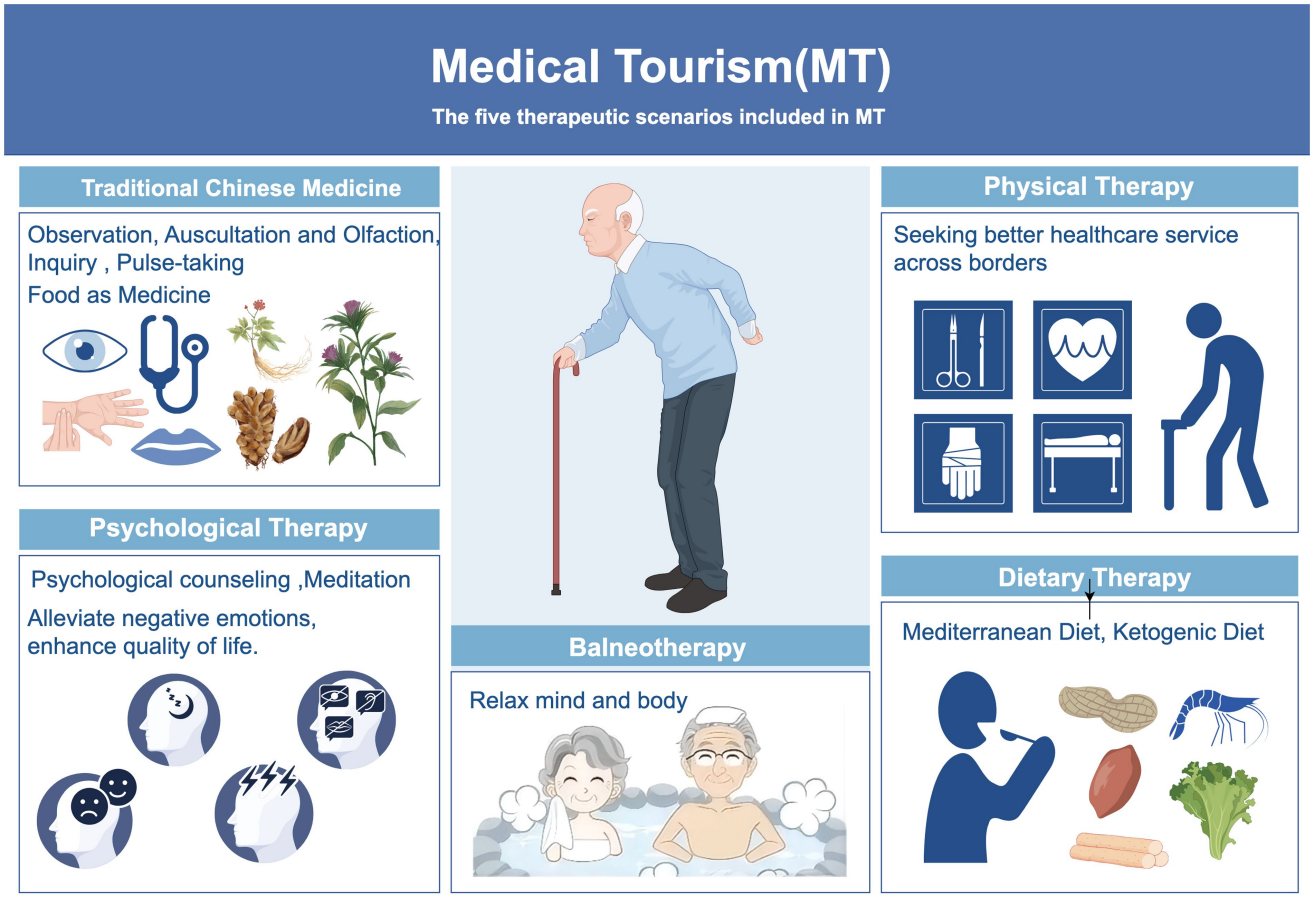
Five application scenarious of MT. MT promotes healthy aging by meeting older adults’ diverse health needs through various applications. Traditional Chinese medicine supports chronic disease management and mind–body balance, while balneotherapy aids relaxation and pain relief. Physical therapy addresses musculoskeletal issues through rehabilitation, and psychological therapies help manage mental health challenges like depression and loneliness. Dietary therapy optimizes nutrition with approaches such as the Mediterranean diet. These integrated methods encourage active health management, supporting longevity and improved QoL. By Figdraw.

The specific content included in MT is shown in the Figure 2.

1. TCM is increasingly integrated into MT, focusing on maintaining overall health and enhancing disease resistance through natural therapies (62–64). TCM employs diverse methods, including mind–body practices like qigong and tai chi, and physical therapies such as acupuncture, moxibustion, and herbal medicine, to achieve balance and prevent illness. Emphasizing the holistic health concept of “treating pre-disease,” TCM prioritizes health cultivation and prevention, making it highly relevant in MT (41, 65). Studies recommend incorporating TCM services, such as acupuncture, tuina, qigong, and tai chi, into health management plans for chronic disease management (e.g., arthritis, hypertension, diabetes) and postoperative recovery (66–70). Acupuncture, in particular, has shown significant efficacy in relieving chronic pain and aiding postoperative recovery (71–73). Additionally, TCM’s emotional therapy combined with mental health interventions demonstrates positive effects in treating depression, anxiety, and preventing Type 2 diabetes (74–77). This comprehensive approach addresses both physical symptoms and mental well-being, offering patients a more holistic health management solution.
2. Physical Therapy (PT) is a key component of MT, playing a critical role in postoperative rehabilitation and older adult health management (78–80). International medical tourists often prefer countries offering high-quality PT services at lower costs, such as Thailand, India, Japan, and South Korea. These destinations attract patients with advanced techniques in manual therapy, exercise therapy, and neurorehabilitation (51, 80–82). Additionally, countries like India, Thailand, and Singapore have become popular MT hubs due to their state-of-the-art healthcare facilities and competitive pricing (80). By integrating modern medicine with traditional therapies, they offer diverse rehabilitation options. Notably, the integration of traditional Chinese massage (tuina) with PT in comprehensive rehabilitation programs has gained traction in some MT institutions. This innovative approach is particularly effective for spinal disorders, arthritis, and chronic pain management, showcasing the benefits of combining Eastern and Western medicine (83–85). Such programs enhance treatment outcomes while providing more personalized health management solutions for patients.
3. Mental health has emerged as a critical focus within the realm of MT, with psychological therapies playing an increasingly important role in addressing anxiety, depression, and stress management (86). Popular MT destinations such as India and Japan have incorporated various psychological interventions into their service offerings, including mindfulness therapy, cognitive behavioral therapy (87), and meditation programs, providing comprehensive support for tourists’ mental well-being (88–91). Moreover, the integration of TCM’s emotional therapy with modern psychological approaches has demonstrated unique advantages in MT programs. This hybrid model has been widely applied to provide psychological support for cancer patients and individuals with chronic diseases, effectively alleviating negative emotions and improving overall QoL (74–77). By combining traditional medical wisdom with contemporary psychological techniques, this innovative approach not only enriches the service portfolio of MT but also offers new solutions for global mental health management.
4. Balneotherapy, as a key component of MT, has demonstrated significant value in rehabilitation and relaxation. It is widely used in the treatment of rheumatic diseases, skin conditions, cardiovascular issues, and post-surgical recovery, offering patients diverse forms of health support (58, 60, 92–94). For instance, countries such as Hungary, Spain, and Japan have integrated mineral-rich hot springs, hydrotherapy, mud baths, and aromatherapy to create comprehensive wellness experiences for visitors (95–97).

Additionally, Beijing’s Xiaotangshan Hot Spring Therapy in China stands out as a classic global MT case due to its long history and unique therapeutic effects. According to historical records, the Xiaotangshan hot spring water is classified as a high-temperature, fluoride-containing, bicarbonate-calcium-sodium, and mild thermal mineral spring with notable medical and health benefits. The Xiaotangshan Hospital utilizes this natural resource to provide specialized aquatic rehabilitation treatments for patients and hydrotherapy programs for sub-health populations. These include water-based exercise rehabilitation techniques (such as Halliwick and Bad Ragaz methods), whirlpool bubble baths, limb electro-hydrotherapy, steam therapy, and herbal baths (98).

Research indicates that balneotherapy not only effectively alleviates chronic pain but also plays a positive role in improving post-COVID-19 symptoms while promoting psychological relaxation and overall well-being (95, 99). By combining natural elements with modern medical technologies, this therapy offers innovative pathways for global health management and enriches the service offerings of MT. Whether at internationally renowned spa destinations or local cases like Xiaotangshan, balneotherapy highlights its unique advantages in enhancing patients’ QoL.

Nutrition plays a critical role in aging and health, with dietary imbalances and deficiencies in essential nutrients closely linked to the aging process (100–103). In recent years, nutritional supplements have garnered significant attention due to their potential as key contributors to longevity and providers of antioxidant molecules. As dietary supplements, these nutrients demonstrate considerable promise in slowing or even halting the aging process and are increasingly being integrated into daily diets for their long-term health benefits (102, 104, 105). Furthermore, dietary supplements and natural compounds such as resveratrol and curcumin are considered dietary restriction mimetics that help delay aging and reduce the risk of chronic diseases (Alzheimers Diseases, Obesity, Cardiovascular Diseases, Type-2 Diabetes) (106–109). Natural therapies derived from medicinal plants and their products are also widely used in disease treatment, regulation, and prevention (110). In this context, Dietary Therapy has emerged as a core non-pharmacological intervention in chronic disease management. For example, diets such as the Mediterranean diet and anti-inflammatory diets, combined with the use of natural compounds, not only promote metabolic health but also enhance cellular resistance to aging, becoming an integral part of health management (111). Multiple studies have shown that Mediterranean diet interventions help participants develop healthy eating habits, significantly improving both physical and mental well-being (112, 113). This dietary pattern is particularly effective in managing conditions such as diabetes, cardiovascular diseases, and digestive system disorders.

Although the MT sector already includes various forms such as TCM, physical therapy, psychological therapies (114), and balneotherapy (e.g., mud baths) (45, 64, 89, 95), specialized travel aimed at accessing dietary therapy has yet to be fully developed. Currently, dietary therapies like TCM medicinal cuisine, Zen diets, and Mediterranean diet interventions are primarily applied in specialized treatment settings (112, 113) rather than serving as a core attraction within MT. As MT transitions from single-focused medical services to comprehensive health management, integrative non-pharmacological interventions combining Eastern and Western medicine offer tourists a more personalized and sustainable health experience. In the future, the MT industry can better meet the diverse needs of global medical tourists, particularly older adult travelers concerned with chronic disease management, rehabilitation, and healthy aging, by optimizing standard systems for non-pharmacological therapies, enhancing service infrastructure, and strengthening cross-cultural medical collaborations. This approach not only enriches the MT landscape but also provides innovative solutions to address the growing demand for holistic health care.

### 2.2 Healthy aging

**Figure 3:**
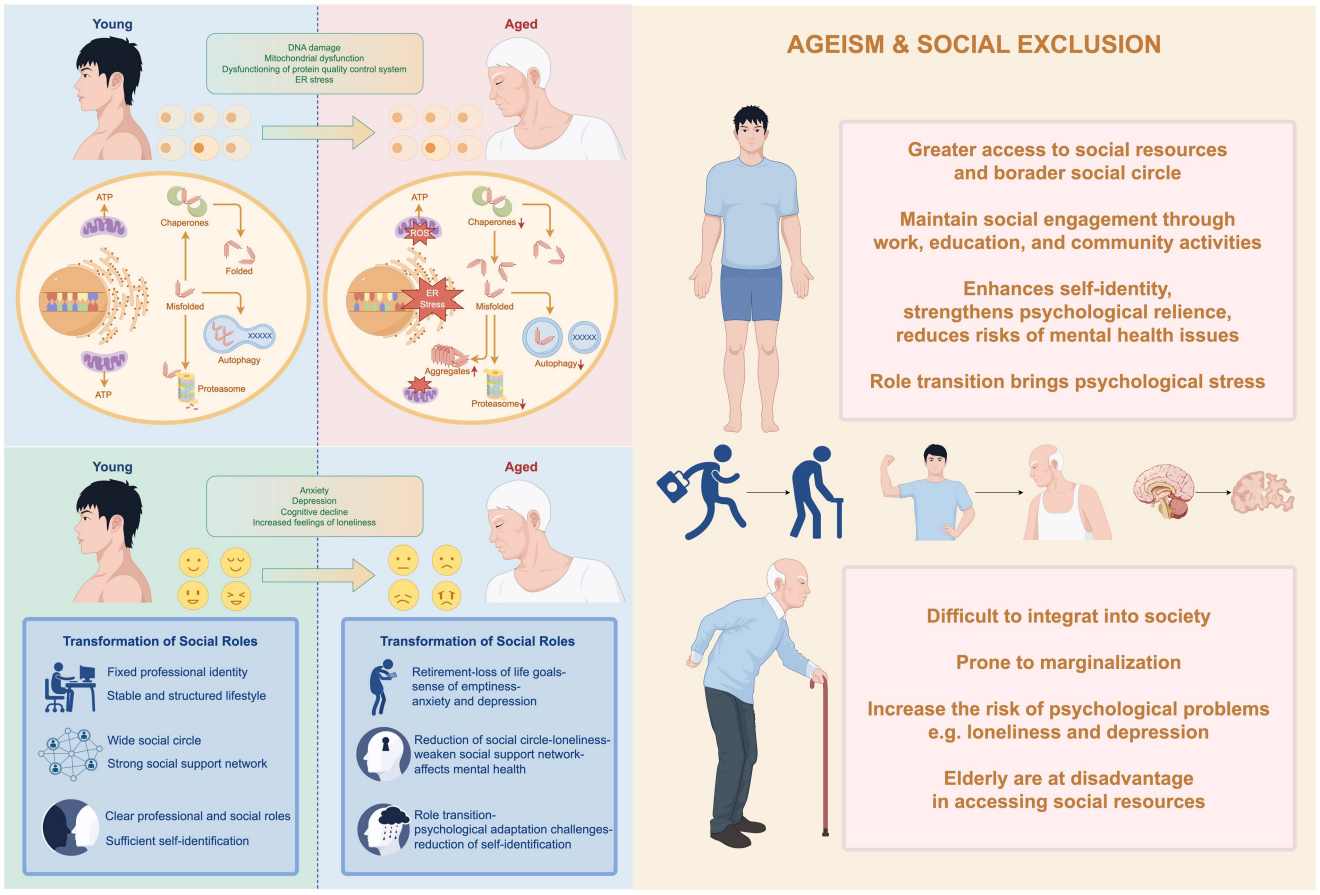
Detailed schematic illustration of the mechanistic pathways of aging processes. Aging is a multidimensional and complex process that encompasses physiological, psychological, and social dimensions. At the physiological level, aging is accompanied by changes such as genomic instability, telomere shortening, loss of proteostasis, and mitochondrial dysfunction, which collectively contribute to the decline in organismal function. At the psychological level, older adults may face challenges such as cognitive decline, increased risks of depression, anxiety, and loneliness, issues that are often closely linked to the transition of social roles. At the social level, against the backdrop of global population aging, problems such as ageism and social exclusion have become more pronounced. These issues not only hinder the social integration of older adults but also exacerbate their psychological burdens, creating additional barriers to well-being and QoL. Addressing these multifaceted challenges requires integrated approaches that consider the interplay between biological, mental, and societal factors, promoting healthy aging through comprehensive interventions and supportive policies. By Figdraw.

**Figure 4:**
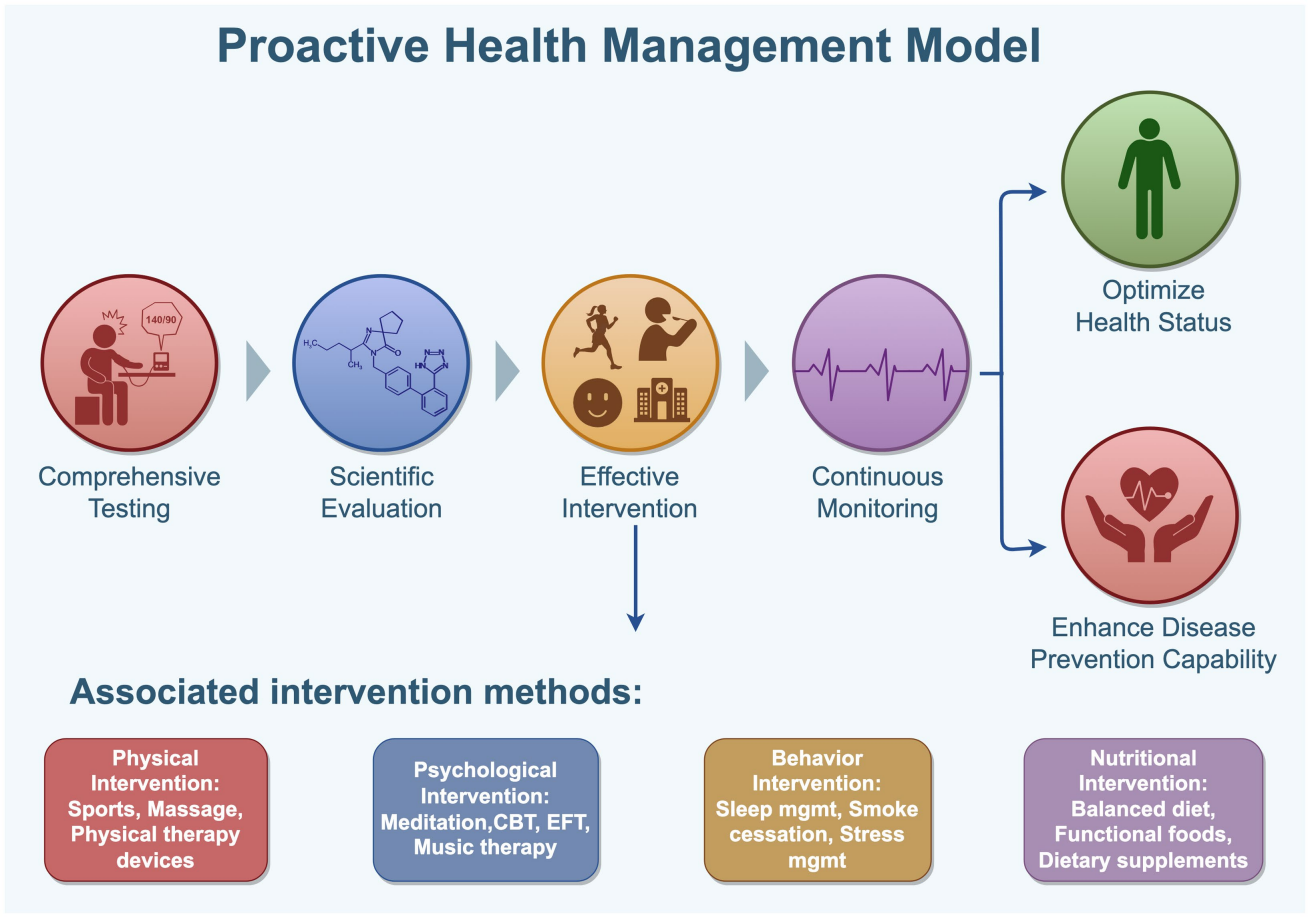
Proactive health management model. Proactive health is a key strategy to advance preventive care and meet the growing health needs of the population. It addresses the limitations of passive and reactive healthcare by encouraging individual agency, promoting self-awareness, early detection, and active problem-solving, thereby reinforcing personal responsibility for health. This approach emphasizes non-pharmacological interventions such as physical, psychological, behavioral, and nutritional methods, fostering healthy lifestyles, balanced diets, regular exercise, positive mental health, and timely disease prevention. These efforts aim to improve health literacy, enhance disease resistance, delay aging, and promote longevity. By Figdraw.

Understanding the biological mechanisms of aging and its impact on individual function is a critical starting point for exploring strategies for healthy aging and extending healthy life expectancy. Aging is a progressive biological process driven by multiple factors, marking the transition of an organism from its mature phase into a state of senescence (115). This process induces widespread functional changes across organs and tissue systems, significantly increasing the risk of age-related diseases and mortality. Aging affects all levels of biological complexity, from molecular to systemic, leading to a comprehensive decline in physiological function and making individuals more susceptible to chronic diseases and other health issues (116, 117). Its core characteristics include degenerative physiological changes in tissues and organs, which not only weaken the body’s self-repair capabilities but also markedly increase vulnerability to various chronic conditions, ultimately culminating in the end of life (116, 117). At different biological levels, aging manifests through a diverse array of features, as illustrated in Figure 3. These features highlight how aging impacts cellular integrity, molecular stability, and systemic functionality, contributing to the overall decline in health and well-being observed during the aging process. Understanding these mechanisms is crucial for developing interventions aimed at extending healthy lifespan and improving QoL in older adults (see Figure 4).

1. Physiological Level: Aging is accompanied by various changes at the molecular and cellular levels, including genomic instability, telomere attrition, loss of proteostasis, mitochondrial dysfunction, and cellular senescence (116). These changes collectively contribute to the gradual decline in physiological function, manifesting as a series of health issues such as decreased immunity, muscle loss (sarcopenia), osteoporosis, and neurodegenerative diseases. Among these, neurodegenerative diseases pose a significant threat to the quality of life in older adults due to their high prevalence and irreversible nature (118, 119).
2. Psychological Level: As age advances, older adults may encounter several mental health challenges, including cognitive decline, depression, anxiety, and increased loneliness (120). The occurrence of these psychological issues is closely associated with multiple factors, among which the transformation of social roles plays a critical role. For instance, changes in lifestyle after retirement and the shrinking of social circles may weaken an individual’s social support network, thereby further exacerbating the onset and development of mental health problems (121). This dynamic interplay not only affects the psychological state of older adults but also has profound negative implications for their overall QoL.
3. Social Level: In the context of global population aging, issues such as ageism and social exclusion have increasingly become focal points of societal concern (104). These issues hinder older adults’ social integration and may further aggravate mental health problems. However, research indicates that social participation is one of the key pathways to achieving healthy aging (11). By maintaining active social interactions, older adults can effectively reduce mental health risks, enhance subjective well-being, and improve their QoL.

In addition, when evaluating the aging process, lifespan is often used as a proxy indicator. “Chronological age,” which refers to the time elapsed from birth to a specific point in time (122), is commonly employed in this context. However, this concept has significant limitations, as it is more influenced by age-related pathological factors rather than providing a comprehensive reflection of the physiological decline process (115). In contrast, “biological age” serves as a more precise measure and is defined as “the duration an individual can maintain health without major diseases or functional impairments” (123). This concept integrates physical, psychological, and social characteristics of the individual, offering a more accurate representation of the true progression of aging and its impact on health status (122).

Since the 20th century, global life expectancy has significantly increased, rising from approximately 66.8 years in 2000 to 73.1 years in 2019, primarily attributed to advancements in medical technology and continuous improvements in living environments (124). However, with the rapid growth of the aging population, the high prevalence of chronic diseases and age-related illnesses has emerged as a significant societal challenge. Later life is particularly critical for conditions such as cancer, cardiovascular diseases, and neurodegenerative disorders, highlighting prominent health concerns (125). Statistics reveal that over 80% of individuals aged 65 and above suffer from at least one chronic disease, while 68% experience multimorbidity, or two or more concurrent diseases (126). These specific pathologies may independently prove fatal or accelerate mortality risk through combined effects (115). This health burden not only leads older adults to endure prolonged periods of declining QoL due to organ dysfunction and increased disease load but also places substantial pressure on social healthcare resources and personal well-being (127). In response to this challenge, the concept of “healthy aging” has emerged. The World Health Organization defines healthy aging as “the process of developing and maintaining functional ability that enables well-being in older age,” emphasizing functional capacity across various domains, including meeting basic needs, learning and growing, mobility, social interaction, and societal contribution (128). Unlike merely extending lifespan, healthy aging focuses on healthspan—the duration during which an individual lives free of major diseases or disabilities (123). By optimizing daily functional abilities and enhancing QoL, healthy aging aims to intervene in and improve the healthspan of individuals (104). Notably, behavioral, lifestyle, and physical environmental factors—proximal risk factors—are modifiable compared to distal factors like genetics and social environment (129), making them prime targets for intervention.

Thus, healthy aging represents not only a goal but also a feasible process (104). Current interventions include pharmacological and non-pharmacological approaches; the former targets disease prevention and treatment, while the latter enhances health and well-being through physical activity, nutritional adjustments, social interactions, and positive psychological interventions (125, 130). Given their cost-effectiveness and minimal side effects, non-pharmacological interventions are prioritized. Over the past decade, there has been a rapid increase in interventions supporting active aging, with notable progress in physical activity, chronic disease self-management, healthy eating, and social functioning (131). Studies indicate that physical activity plays a crucial role in maintaining community-based QoL among older adults (132, 133). Additionally, participation in daily activities and meaningful social practices is considered essential for healthy aging (134). Traditional plant-based foods, rich in bioactive compounds, have demonstrated unique value in healthy aging. Research by Rajaram et al. shows that these natural components reduce oxidative stress, lower inflammatory responses, and promote cellular repair, collectively slowing the aging process (135). Furthermore, MT serves as a comprehensive intervention approach, integrating high-quality medical resources, rehabilitation therapies, balneotherapy, exercise rehabilitation, mental health interventions, TCM massage, and religious participation. This multifaceted strategy not only reduces chronic disease risks and improves functional capacity but also alleviates loneliness by enhancing social interactions and overall well-being (136).

Nevertheless, current research on aging and MT remains relatively fragmented, primarily focusing on areas such as cross-border surgical procedures, travel-based rehabilitation care, and healthcare services (59). Only a limited number of studies have explored the specific benefits of MT in the context of healthy aging, including improvements in health and well-being through tourism for older adults (137), support for chronic disease management (138), enhancement of well-being (139), and increased accessibility to healthcare facilities (140). Goh et al. (141) further highlighted that the health benefits of travel contribute to maintaining functional capacity among older travelers, thereby enhancing well-being during later life stages. Exploring solo travel in later life through a tri-factor healthy ageing framework. DeMicco (142) emphasized the integration of hospitality with healthcare, advocating for the concept of “putting heart back into healthcare” to improve community health and well-being while expanding accommodation revenue. Additionally, Majeed et al. (9) found that MT holds significant implications for aging societies, particularly in the face of growing chronic disease threats. The combination of homeopathy, naturopathy, and TCM offers new possibilities for healthy aging. In summary, while existing research has begun to uncover the potential role of MT in promoting healthy aging, its underlying mechanisms and implementation pathways require further investigation. Future studies should focus more sharply on how MT can effectively enhance the functional capacity and overall well-being of older adults by integrating diverse interventions, ultimately achieving genuine healthy aging.

### 2.3 Proactive health management

Proactive Health Behavior refers to an individual’s capacity to actively seek health information and participate in health-related activities. This not only reflects one’s motivation and willingness to adopt and maintain healthy behaviors but also demonstrates a proactive awareness of managing one’s own health (143). Health Management, on the other hand, is a personalized intervention model based on individual health data, encompassing various aspects such as physical examinations, assessments, treatment and rehabilitation, health education, and insurance services. It is primarily used for the prevention and management of chronic non-communicable diseases (144). Proactive Health Management, as a modern medical practice, transcends the limitations of traditional reactive healthcare and standalone health screenings. By incorporating comprehensive testing, scientific evaluation, effective intervention, and continuous monitoring, it optimizes the health status of both individuals and populations, enhances disease prevention capabilities, and boosts overall immune function (145–149). Research has shown that proactive health behaviors enable patients to better manage their conditions, promote disease recovery, and significantly improve health outcomes (150).

In recent years, the rising prevalence of chronic diseases and its growing incidence among younger populations have prompted a shift from the traditional reactive healthcare model (“treatment after illness”) toward proactive health management (151, 152). This concept aligns closely with the “pre-disease treatment” philosophy in TCM, emphasizing the prevention of diseases through the adoption of healthy lifestyles and habits. Numerous studies have demonstrated the significant efficacy of proactive health management in chronic disease prevention and its ability to meet the demand for high-quality health care. For instance, Jiang et al. found that community-based proactive health management apps could enhance electronic health literacy and self-management skills among hypertensive patients, enabling better participation in health management, reducing blood pressure, and improving overall QoL (153). Similarly, Tang et al. analyzed data from interviews with 20 pre-frail older adults in Guangzhou geriatric wards, concluding that active health behaviors are crucial for developing proactive health strategies for this population (40). He and Wang’s study in four Beijing communities revealed strong resident interest in proactive health management and high regard for community healthcare services (41). Furthermore, Yang’s research involving long-term follow-ups of 8,644 chronic disease patients demonstrated that grid-based community health management significantly improved the relevance and coverage of community health services while reducing hospitalization durations for type 2 diabetes patients (13). As a forward-looking health intervention model, proactive health management holds great potential in addressing the growing burden of chronic diseases and enhancing public health. Future research should focus on exploring practical implementation pathways and effective evaluation methods to promote its global application.

The intrinsic motivations driving patient participation in proactive health behaviors primarily include a sense of health responsibility and health needs, while external support also plays a critical role in promoting such behaviors. For example, Zhang et al. found through interviews with middle-aged and older adult women suffering from urinary incontinence and their caregivers that active health practices among these groups mainly involved healthcare behaviors (such as regular medical check-ups and adherence to prescribed medication) and the establishment of healthy lifestyles (such as balanced diets and regular exercise) (150). This study highlights that health management encompasses not only individual-level health behaviors (e.g., healthy eating, diabetes control, moderate exercise) but also relates closely to public health equity, reflecting the importance of fair distribution of medical resources in society (154, 155). Specifically, Yang’s research on the grid-based family doctor community health management model in Shandong Province demonstrated that this mechanism significantly facilitated the implementation of national basic public health services and improved the accessibility of community public health services (13). By precisely tracking and monitoring key populations not yet covered, the model effectively addressed gaps in national basic public health services, further optimizing resource allocation efficiency and service quality. These findings underscore the dual value of proactive health management in enhancing both individual health levels and overall societal well-being. Moreover, existing studies indicate that proactive health management plays an indispensable role in chronic disease prevention and older adult health management, with growing public demand for such approaches. In this context, MT serves as an important practical tool, integrating high-end medical services, rehabilitation, chronic disease management, and health promotion into a comprehensive solution tailored for older adults and individuals with chronic conditions. By combining quality medical resources with personalized health management, MT not only meets individual health needs but also contributes to achieving global healthy aging.

The global health industry encompasses multiple sectors, including medical services, health products, nutrition, health equipment, MT, and health consulting, with its market size continuing to grow (156). Data shows that by 2022, the scale of China’s health industry had exceeded 12 trillion CNY, and it is projected to surpass 29.1 trillion CNY by 2030 (157).

Against the backdrop of increasingly diversified healthcare needs and the rapid advancement of artificial intelligence technologies, the demand for AI-driven innovations among residents, healthcare institutions, and related caregiving personnel has grown exponentially in recent years (158–163). In the healthcare domain, AI-driven decision-making has the potential to advance disease diagnosis, personalize treatment strategies, and optimize resource allocation. AI-enabled decision support in Hospital Information Systems (HISs) plays a critical role by equipping healthcare providers with tools to analyze complex patient data, thereby enabling more accurate and timely clinical decisions (159). Leveraging machine learning algorithms and predictive analytics, AI facilitates early disease detection, treatment adjustments, and efficient resource planning. Its ability to process large volumes of heterogeneous health data allows for the rapid identification of patterns and correlations that may not be apparent through conventional methods, ultimately improving patient outcomes (164). The integration of sensor technologies with AI and machine learning further enhances diagnostic accuracy and supports proactive, data-driven clinical decision-making (163). A growing body of research now focuses on interdisciplinary applications at the intersection of medicine and engineering, including the development of medical AI models (165, 166), large-scale healthcare question-answering systems (167–169), AI-assisted diagnostic and treatment tools (170), as well as AI models for TCM diagnostics (171–173). Emerging technologies such as Internet of Things (IoT), AI, large language models (LLMs), deep learning, machine learning, and continual learning algorithms are being increasingly integrated into healthcare systems, paving new pathways for proactive health management.

### 2.4 Continual learning

Lifelong or continual learning (174–179) refers to the challenge of sequentially acquiring knowledge from non-stationary data streams, where new tasks arrive over time and the input distribution (i.e., concepts) may drift (180, 181). Traditional deep learning models, which assume static datasets, suffer from catastrophic forgetting when exposed to evolving environments (182). This leads to the stability–plasticity dilemma, wherein the model must balance retaining prior knowledge and adapting to new inputs (180).

Many researchers (183, 184) categorized continual learning into three primary scenarios:

Task-incremental learning (Task-IL): Each task is known during training; thus, task-specific model heads can be used.
Domain-incremental learning (Domain-IL): The task remains the same, but data distributions shift without task labels.
Class-incremental learning (Class-IL): Neither task identity nor class boundaries are provided, resembling real-world multi-task health scenarios.

Continual learning frameworks, especially under domain-IL and class-IL, are highly applicable to healthcare data environments characterized by concept drift, multimodal inputs, and progressive personalization needs (180).

In the proposed dynamic health portrait-driven model, continual learning serves as the core mechanism to capture evolving environmental, physiological, and behavioral signals across multiple therapeutic tourism scenarios. Unlike static AI models trained on one-off datasets, our system adapts to individual-level concept drift, using real-time multimodal data from natural non-pharmaceutical interventions (e.g., forest bathing, mineral springs, guided meditation). In such contexts, continual learning addresses the stability–plasticity dilemma by allowing the system to learn new tasks (e.g., stress relief, cardiovascular recovery) without erasing previous knowledge (e.g., sleep regulation), thus forming a self-evolving health profile. Specifically, our model aligns with domain-incremental learning (Domain-IL) and class-incremental learning (Class-IL) settings, enabling the architecture to adjust to changes in intervention types, user needs, and location-based health contexts without re-training from scratch.

## 3 Materials and methods

This study adheres to the PRISMA framework (185) and conducts a systematic review of literature related to MT and healthy aging. The research follows four stages—identification, screening, eligibility assessment, and final inclusion—and employs a systematic literature search methodology to construct a conceptual model. This model aims to explore the potential role of MT in promoting healthy aging. From December 2024 to January 2025, we conducted a literature search in Web of Science, PubMed and CNKI databases, focusing on high-quality journals that address themes of tourism, health, and medicine. The selection criteria included articles published after 1990 in SJR Q1 peer-reviewed journals (186). To ensure the quality and authority of the selected literature, we specifically targeted English-language peer-reviewed articles published in SJR Q1 journals since 1990. The search strategy was structured around the following key themes: MT, Healthy Aging, Older Adult Tourists, Health Benefits of MT, and MT in the Context of Comprehensive Health. Specific keyword combinations were used as follows:

1. Healthy aging-related terms: “healthy aging” OR “positive aging” OR “active aging” OR “robust aging” OR “successful aging” OR “aging well”;
2. MT-related terms: “MT*” OR “healthcare tourism*”;
3. Search terms included “older adults,” “older people,” “aged,” and “elderly.”

**Figure 5:**
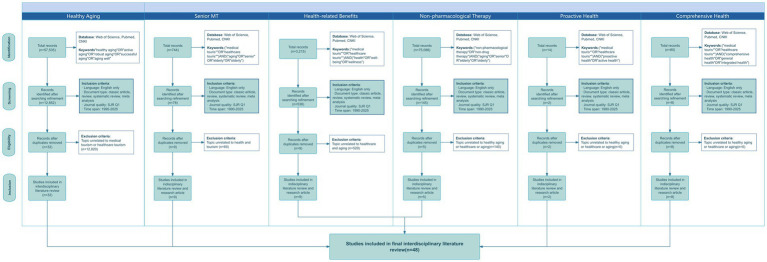
Flowchart of PRISMA-based literature selection process for MT and healthy aging research.

The specific classification and results analysis are presented in Figure 5.

The literature search process began with the identification of articles related to healthy aging using keywords such as “healthy aging,” “positive aging,” “active aging,” “robust aging,” “successful aging,” and “aging well.” This initial search yielded 57,535 articles, of which 12,852 met the predefined inclusion criteria. Among the interdisciplinary studies identified, only 16 were found to be relevant to tourism, healthcare, and medicine, with 13 specifically addressing MT. Using a combination of keywords (“MT*”) or (“healthcare tourism*”) along with (“elderly” or “older adults” or “senior” or “aging”), 744 publications were retrieved, from which 78 papers were selected. However, most of these articles focused on MT for general tourists in the fields of medical and cosmetic procedures, with only nine mentioning population aging. None of these addressed health-related retirement issues. An additional search using the keywords (“MT*”) or (“healthcare tourism*”) combined with (“health” or “well-being” or “wellness”) resulted in 3,215 files, of which 538 were retained. These studies primarily centered on general tourists and residents, with only nine articles touching upon health, care, and aging. None explored the role of MT in promoting healthy aging. A further search using the keywords (“MT*”) or (“healthcare tourism*”) combined with (“comprehensive health” or “general health” or “integrated health”) yielded 80 articles, of which eight were retained. However, these studies focused on the broader context of the health industry, targeting the general population rather than older adults, aging, or healthy aging. Using the keywords (“MT*”) or (“healthcare tourism*”) combined with (“proactive health” or “active health”), 14 relevant articles were identified, but only two met the screening criteria. Unfortunately, these two articles focused on public health in India and did not address health-related retirement issues. Finally, a search using the keywords (“NPT”) or (“non-drug therapy”) combined with (“elderly” or “older adults” or “senior” or “aging”) retrieved 75,088 articles, of which 145 met the inclusion criteria. Among these, only two studies mentioned health-related retirement, two focused on mental health aspects of geriatric depression, and one addressed cognitive disorders in older adults. Based on the above literature search and screening process, this study ultimately included 48 articles for an in-depth review.

## 4 The role of MT in managing chronic conditions among older adults

According to the World Health Organization’s (WHO) Global Strategy and Action Plan on Ageing and Health 2016–2020, the second strategic goal is to create age-friendly environments through multi-sectoral collaboration, integration across multiple settings, and active participation of older people (187). In this context, the tourism industry, particularly MT, can play a critical role in promoting healthy aging. Studies show that environmental factors and lifestyle have a more significant impact on healthy aging than genetic factors and can be optimized through non-pharmacological interventions such as physical activity, social interaction, nutritional improvement, and mental health management (125, 128, 130). MT not only inherits the health benefits of traditional travel but also provides comprehensive health support for the older adults through precision medical services, personalized health management, and rehabilitation interventions, particularly through the application of key intervention methods such as traditional therapies (e.g., Tai Chi, massage), hydrotherapy, physical therapy, psychological intervention, and dietary adjustment. Its core functions are specifically focused on optimizing the following five dimensions of health management: Physical activity, nutrition, social interaction, emotional health, and physical therapy.

### 4.1 Physical activities and rehabilitation in MT

Physical activity is the fourth leading risk factor for mortality among older adults, significantly increasing the likelihood of frailty, chronic diseases, and disability (188, 189). Research indicates that regular participation in physical activity not only substantially reduces the risk of functional impairments but is also considered one of the most cost-effective lifestyle interventions (128). For older adults, maintaining physical capability is not only a critical foundation for independent living but also a key strategy for disease prevention and delaying aging. Ordinary travel inherently involves various forms of natural movement, such as walking, hiking, cycling, and swimming, which help older adults preserve muscle strength, joint flexibility, and cardiovascular health, thereby sustaining their mobility and independence (190, 191). MT further expands the possibilities of exercise rehabilitation. For instance, postoperative recovery, physical therapy, and functional training offer personalized exercise support tailored to the specific health needs of older adults (192). Furthermore, MT integrates multiple NPT from TCM, including acupuncture, moxibustion, qigong, tai chi, and TCM massage. These traditional therapies have been proven effective in promoting blood circulation, relieving muscle tension, and improving nervous system function (66–70). By combining modern medical technologies with traditional rehabilitation methods, MT provides older adults with comprehensive functional restoration solutions, helping them regain mobility and enhance their QoL.

Research demonstrates that various traditional and modern exercise interventions play a significant role in promoting healthy aging. For example, Linda Yin-King Lee and Eric Chun-Pu Chu found that tai chi not only enhances muscle strength and balance but also alleviates stress and improves mood, offering both physiological and psychological benefits. Its gentle, safe, easy-to-learn, and cost-effective nature makes it particularly suitable for older adults, including residents in care facilities (193). Meanwhile, aging is often accompanied by deteriorating bone health and an increased risk of osteoporosis. Zhuping et al. (194) conducted a systematic analysis and ranking of bone-related studies, comparing the effectiveness of different exercises. They proved that traditional Chinese fitness practices such as tai chi, baduanjin (eight pieces of brocade), yijinjing (sinew Metamorphosis), and wuqinxi (195) significantly improve physical and bone health, effectively preventing osteoporosis (196–198). Furthermore, Wang et al. established a rat model of knee osteoarthritis (KOA) and found that tuina (Chinese therapeutic massage) significantly improved outcomes in KOA rats by regulating chondrocyte apoptosis and autophagy, inhibiting inflammatory cytokine secretion, alleviating inflammation, and promoting cartilage repair (199). This highlights the importance of tuina as a NPT in the prevention and treatment of knee osteoarthritis. Furthermore, by dynamically analyzing data such as gait stability and joint mobility collected from wearable devices (e.g., inertial sensors, foot pressure pads) using continual learning algorithms, rehabilitation plans can be optimized in real time. For example, when the algorithm detects a significant improvement in gait coordination in older adult patients during hot spring therapy, it will automatically recommend extending the aquatic exercise program or switching to mountain hiking therapy; Conversely, if balance ability fluctuates, the exercise intensity is promptly reduced and the frequency of traditional Chinese massage is increased. This dynamic adjustment mechanism based on incremental learning avoids the “catastrophic forgetting” caused by data updates in traditional models, significantly enhancing the precision and timeliness of rehabilitation interventions. Kyung Eun Lee et al., using the PRISMA standard for systematic reviews and meta-analyses, analyzed 21 reports on rehabilitation interventions for gout patients. Their findings indicated that aerobic exercise and home-based exercise programs significantly aid in post-stroke rehabilitation, improving patients’ motor abilities and QoL (200). Patti et al. conducted exercise training studies on heart failure patients, showing that cardiac rehabilitation not only enhances patients’ exercise capacity and QoL but also effectively reduces depressive symptoms, improves survival rates, and lowers hospitalization risks (201), further confirming the critical role of exercise rehabilitation in chronic cardiovascular disease management. Hoshino pointed out that dialysis patients exhibit significantly lower levels of physical activity compared to the general older population (202). However, tracking dialysis patients who underwent exercise interventions revealed that exercise significantly improves their physical capabilities and QoL, which are two core components of renal rehabilitation. Lastly, Keteyian et al. systematically reviewed exercise testing and rehabilitation data for atrial fibrillation patients, finding that exercise rehabilitation not only enhances patients’ exercise capacity and QoL but may also alleviate atrial fibrillation-related symptoms (203). These studies collectively demonstrate that both traditional Chinese fitness practices and modern exercise interventions have significant effects in promoting healthy aging. These interventions not only improve physical function in older adults but also enhance mental health and social engagement, providing important practical pathways toward comprehensive healthy aging (see Table 1).

**Table 1:** Analysis of the role of physical activity as a NPT in healthcare.

Intervention	Region	Sample	Health Focus	Key Findings	Source
Tai Chi	China	Nursing home resident	Healthy aging	Tai Chi has been shown to lower blood pressure, reduce resting heart rate, decrease cholesterol levels, alleviate joint pain, and mitigate symptoms of depression and anxiety, all of which contribute positively to healthy aging.	Lee and Chu (193)
Traditional Chinese fitness exercises	China	Bone Mineral Density (BMD) in middle-aged and older adult populations	Osteoporosis	Traditional Chinese fitness exercises have potential advantages in improving bone mineral density (BMD) across various parts of the body.	Zhuping Ren and Liyue He and Xiaoran Li and Lingxiang Yan and Zhuying Ren and Xiaolei (194)
Tuina	China	Sprague Dawley rats	KOA	Tuina can alleviate cartilage tissue damage in KOA, reduce inflammation, and decrease chondrocyte apoptosis and autophagy.	Wang et al. (199)
Rehabilitation exercises	Korea	Stroke patients	Stroke	Compared to single forms of exercise, complex or combined exercises are more effective for the rehabilitation and treatment of gout. In the early stages of a stroke, physical exercise rehabilitation is required instead of bed rest; in the late acute phase, aerobic exercise and home-based exercises are necessary; during the chronic stage, a combination of exercise programs and remote rehabilitation using smart devices is needed.	Lee et al. (200)
Personalized exercise programs, including aerobic training, strength training, flexibility training, and/or inspiratory muscle training.	Italy	Heart failure patients	Heart failure	Exercise training has comprehensive benefits for patients with heart failure. Exercise interventions can improve patients’ exercise capacity, alleviate depressive symptoms, increase survival rates, and reduce the risk of hospitalization.	Patti et al. Patti et al. (201)
Exercise interventions, including aerobic exercise, resistance training, balance training, and flexibility exercises.	Japan	Chronic kidney disease patients, Dialysis patients	Chronic kidney disease	The organic combination of four different types of exercise intervention modes has significant effects on the rehabilitation, physical health, and QoL of dialysis patients; however, dialysis patients tend to avoid exercise.。	Hoshino (202)
Exercise training, including aerobic exercise and strength training.	America	Atrial fibrillation patients	Atrial fibrillation	With exercise training, the duration of atrial fibrillation decreases, the average ventricular rate declines, the atrial fibrillation burden is reduced, and the arrhythmia-free survival rate improves.	Keteyian et al. (203)

### 4.2 Nutrition and dietary management in MT

Aging is often accompanied by physiological changes in older adults, such as difficulty in chewing, diminished taste perception, and declining digestive function, which may lead to reduced appetite, malnutrition, and weight loss (128, 204). Chronic malnutrition over time can accelerate muscle loss, cognitive decline, and immune system impairment, further affecting the QoL of older adults and significantly hindering healthy aging (129). Therefore, scientifically sound nutritional and dietary management plays a crucial role in promoting the health of older adults. Travel, as a lifestyle intervention, offers older adults opportunities to experience diverse and healthy diets. For instance, the Mediterranean diet, rich in fish, olive oil, and whole grains, has been shown to effectively improve cardiovascular health (205). MT further integrates personalized dietary plans, nutritional counseling, and metabolic testing into the health management process, providing older adults with more comprehensive support. Studies indicate that traditional plant-based foods contain abundant bioactive compounds, which, through mechanisms such as antioxidation, anti-inflammation, and cell regeneration, can effectively slow down the aging process (206). Specifically, these natural components reduce oxidative stress, lower inflammatory responses, and promote cellular repair, playing an important role in achieving healthy aging.

For example, Li et al. conducted a questionnaire survey of 3,982 older adults in Chinese communities and calculated their dietary quality index, finding that higher dietary quality significantly reduced the risk of all-cause mortality and cancer-related mortality associated with underweight or obesity in older adults (207). Additionally, anti-inflammatory and antioxidant diets have been shown to play an important role in preventing cardiovascular disease mortality related to obesity (207). Gross et al. studied 7,300 eligible older adults to evaluate the relationship between frailty status and adherence to the Mediterranean diet, with results indicating that the Mediterranean diet significantly reduces frailty in older adults (105). It is worth noting that the continuous learning algorithm can deeply integrate local dietary databases with patients’ personalized needs. For example, during MT in Thailand, the algorithm analyzes real-time blood glucose monitoring data and dietary logs to dynamically learn patients’ metabolic responses to tropical fruits, generating adaptive recipes: recommending low-GI local fruits (such as pomelo instead of mango) paired with high-fiber palm heart salad, preserving regional dietary characteristics while ensuring stable blood glucose levels. The algorithm employs an incremental training mechanism to continuously integrate ingredient characteristics from different wellness destinations and patient feedback, gradually building a cross-cultural nutritional intervention knowledge base. Giordano et al., through a systematic review of literature and data analysis, found that the Mediterranean diet effectively lowers the incidence of geriatric cancers, though its specific impact on cancer mortality requires further investigation (208). Veronese et al. pointed out that due to its rich nutrients and bioactive compounds, the Mediterranean diet is an effective method for preventing and managing osteoarthritis, promoting rehabilitation, or preventing disease progression (209). Furthermore, Ma et al. analyzed the relationship between activities of daily living (ADL) and basic activities of daily living (Ma et al.) in older adults and their tea-drinking habits using data from the 2008–2018 Chinese Longitudinal Healthy Longevity Survey, showing that tea consumption has a protective effect against disability in older adults (210). Şimşek and Uçar, through a systematic review of literature, explored the application of the ketogenic diet in Alzheimer’s disease (AD) or mild cognitive impairment, demonstrating that the ketogenic diet effectively protects or improves cognitive function in older adults (211). Lastly, Sakatani et al. developed a cognitive function screening test based on deep neural networks and evaluated older adults in gyms, revealing that therapy combining exercise and diet significantly improves cognitive function in older adults (212). These studies highlight the significant role of proper dietary structure and lifestyle interventions in promoting the health of older adults, preventing chronic diseases, and improving cognitive function. By optimizing dietary quality, adhering to the Mediterranean diet pattern, integrating exercise and nutritional therapies, and exploring personalized dietary solutions such as the ketogenic diet, it is possible to effectively enhance the QoL and overall health of older adults (see Table 2).

**Table 2:** Nutrition and dietary management as a non-pharmacological intervention in healthcare.

Intervention	Region	Sample	Health focus	Key Findings	Source
Healthy Diet Patterns: Oxidants, Vitamins, Minerals, and Fiber	China	3,982 community-dwelling older adults	Weight issues, cancer, obesity-related cardiovascular diseases	A balanced diet rich in antioxidants, vitamins, minerals, and fiber reduces all-cause and cancer mortality risks in older adults with weight problems. It provides comprehensive nutrition, supports physical health, and mitigates cancer risk and progression.	Li et al. (207)
Mediterranean Diet	America	7,300 older adults aged 60+	Frailty	The Mediterranean diet plays a potential role in maintaining physical strength and delaying or reducing frailty progression.	Gross et al. (105)
Mediterranean Diet	Italy	Older adults aged 60+	Cancer	The Mediterranean diet exhibits significant anti-inflammatory and antioxidant properties, reducing cancer incidence in older adults but showing less clear effects on cancer mortality.	Giordano et al. (208)
Mediterranean Diet	Italy	Older adults with OA	OA	The Mediterranean diet’s strong antioxidant and anti-inflammatory capabilities help control metabolic abnormalities, maintain weight, bolster immune system resilience, and alleviate OA symptoms.	Veronese et al. (209)
Tea	China	Chinese Longitudinal Healthy Longevity Survey (2008–2018)	Disability in older adults	Regular tea consumption prevents ADL disabilities in older adults. Daily tea drinking lowers the risk of BADL disabilities, though further research is needed to determine optimal dosage.	Ma et al. (80)
Ketogenic Diet	Turkey	Patients with AD and mild cognitive impairment	AD and mild cognitive impairment	The ketogenic diet demonstrates neuroprotective effects, safety, and sustainability, aiding in protecting or improving cognitive function in AD patients. However, long-term adherence may pose certain risks for older adults.	Şimşek and Uçar (211)
Exercise-Based Dietary Therapy	Japan	Older adults at gyms	Vascular dementia and AD-related cognitive impairments	Cognitive function assessed through DL-based tests improves with exercise dietary therapy, which is effective in preventing and managing cognitive disorders in older adults.	Sakatani et al. (212)

### 4.3 Social interaction and psychological health in MT

Moreover, social interaction and mental health play a crucial role in the overall well-being of older adults. Social isolation and loneliness have emerged as significant challenges in global public health. Statistics indicate that approximately 50% of individuals aged 60 and above experience social isolation, while one-third of older adults report feeling lonely (213, 214). Non communicable diseases (215) not only substantially increases the risk of psychological disorders such as anxiety, depression, and dementia but can also lead to chronic conditions like cardiovascular diseases (216). Therefore, promoting social participation among older adults is essential for preventing mental health issues and cognitive decline (11, 217, 218). Travel, as a form of social activity, offers older adults opportunities to meet new people and expand their social networks. During travel experiences, older adults can enhance their social skills and improve their social health through interactions with hosts, fellow travelers, and service providers (219, 220). In particular, within the context of MT, destination centers often provide a range of services such as psychological counseling, meditation, mindfulness therapy, and TCM-based emotional therapies (74–77). These interventions not only help alleviate stress and enhance well-being but also effectively address mental health issues arising from loneliness. By integrating these social and mental health-focused interventions into the travel experience, MT provides a holistic approach to promoting healthy aging, fostering both physical and psychological resilience in older adults.

For example, Yang et al. conducted a data modeling analysis to examine the relationship between social activities, depression, and cognitive function in older adults. The study found that social activities can effectively reduce depressive symptoms by strengthening social connections and providing emotional support. Furthermore, cognitive health interventions not only help protect cognitive function in older adults but also play an important role in preventing and alleviating depression, thereby promoting healthy aging (218). Yin et al. employed a phenomenological approach to conduct an in-depth study of older adults with mild cognitive impairment (MCI). The results showed that participants who sought help from family members and maintained neighborhood social ties not only expanded their mobility within the community but, in some cases, extended it beyond community boundaries. This increased mobility had a positive impact on the rehabilitation of cognitive impairments (221). Notably, during MT, social interactions with family members or strangers can also protect cognitive function by expanding mobility. Soo Yeon Yoo et al. performed a systematic search and detailed analysis of relevant literature, finding that as the frequency of social interactions increases along with significant improvements in cognitive function, the level of depression in older adults decreases significantly (222). Chan et al. conducted a systematic review of six databases, highlighting that mindfulness therapy, as a low-cost and scalable psychological intervention, can be easily integrated into caregiving practices, significantly enhancing the well-being of older adults with MCI and improving their sleep quality (223). Haudry et al. assessed 27 older adult meditation experts and 135 non-meditators, revealing that meditation can reduce negative psychological factors while enhancing positive emotions through specific mechanisms. Additionally, meditation practice can counteract brain volume loss associated with aging, promoting healthier aging processes (224). Hung et al. conducted a survey of older adults receiving care services at “Senior Activity Centers” in Singapore using both qualitative and quantitative research methods. The study demonstrated that psychological counseling not only enhances overall well-being but also effectively boosts dignity among older adults (225). Lastly, Yang et al. conducted a systematic review of database literature and related controlled experiments, finding that TCM-based emotional therapy has significant effects in improving post-stroke depression symptoms. This therapy is advantageous due to its cost-effectiveness, safety, minimal side effects, and ease of acceptance by patients, providing critical support for rehabilitation (77). In conclusion, social activities, psychological interventions, and traditional medicine therapies demonstrate multifaceted potential in promoting mental health and cognitive function in older adults. By strengthening social connections, providing emotional support, and adopting personalized interventions, these approaches not only effectively alleviate depressive symptoms but also offer important support for achieving healthy aging (see Table 3).

**Table 3:** Social interaction and mental health as a non-pharmacological intervention in healthcare.

Intervention	Region	Sample	Health Focus	Key Findings	Source
Social Activities	China	6,802 adults aged 45 and above	Cognitive function, depression	Social interaction optimizes cognitive benefits and indirectly alleviates depressive symptoms. Cognitive and physical stimulation activities have a significant positive impact on mental health.	Yang et al. (218)
Social Interaction, Including Communication with Family Members and Neighborhood Interaction	China	Older adults with MCI	MCI	Interactions with family members or neighbors enhance adaptability and mobility in older adults, which is beneficial for cognitive function.	Qingqing Yin and Lin Chen and Xupeng Mao and Eva (221)
Social Engagement	Korea	Literature review on older adults and social interventions	Depression, cognitive impairment	Social engagement protects cognitive function in older adults and helps prevent or alleviate depressive symptoms.	Yoo et al. Yoo et al. (222)
Mindfulness meditation	British	Older adults with MCI	Sleep disorders in MCI patients	Mindfulness meditation significantly improves sleep quality, reduces insomnia severity, and enhances overall well-being in older adults with MCI.	Chan et al. (223)
Mindfulness meditation	France	27 older adult meditation experts and 135 non-meditators	Aging and mental health	Long-term meditation promotes structural and functional brain protection during aging and improves psychological and emotional factors.	Haudry et al. Haudry et al. (224)
Psychological counseling	Singapore	Adults aged 65 and above	Healthy aging, dignity	Psychological counseling optimizes psychological and behavioral functions, enhances overall well-being, and preserves the dignity of older adults.	Hung et al. (80)
TCM emotional therapy	China	Seven databases on stroke and TCM emotional therapy-related controlled experiments	Post-stroke depression	TCM emotional therapy improves neurological and cognitive functions in post-stroke depression patients and alleviates anxiety and depressive symptoms.	Yang et al. (77)

### 4.4 Emotional well-being and psychological resilience in MT

The fourth aspect is emotional well-being and psychological resilience. As individuals age, their health tends to decline gradually, making them more susceptible to negative emotions such as anxiety, depression, and anger. These emotions not only negatively impact the nervous system, immune system, and cardiovascular system but can also accelerate the aging process (226–228). Conversely, positive emotions like happiness, serenity, and satisfaction can enhance physiological functioning, thereby promoting healthy aging (229). In the realm of MT, various therapies are widely utilized to support emotional regulation and mental health in older adults. For instance, horticultural therapy (HT), balneotherapy, mud baths, hydrotherapy, and aromatherapy have been proven effective in helping older adults relieve stress and improve their psychological state (96, 97). Additionally, TCM emotional therapy offers a unique approach by adjusting emotions and balancing mind–body harmony, providing older adults with specialized mental health support (95). These comprehensive interventions collectively form an essential component of emotional management within MT, offering holistic support for the mental health of older adults. By integrating these therapies, MT not only addresses physical health but also ensures psychological well-being, ultimately contributing to a higher quality of life during aging.

For example, Zhao et al. conducted a systematic review based on the PRISMA guidelines, analyzing database literature and controlled experiments involving HT for dementia patients. The study found that HT not only protects cognitive function in dementia patients but also effectively induces positive emotions and increases their engagement (230). Additionally, Torres-Prunonosa, Jose et al. modeled and analyzed the economic benefits of spa tourism in Spain. Their results revealed that the primary motivations for spa tourists included relaxation (50.16%), recovery (28.20%), followed by curing illnesses (13.77%), disease prevention (5.90%), and leisure (1.97%) (97). These findings highlight the significant potential of hydrotherapy as a non-pharmacological intervention in promoting both mental and physical health. Liu ZY et al. conducted a randomized controlled trial involving 44 office workers to evaluate the effects of aromatherapy, with objective analysis performed using electroencephalography. The results showed that aromatherapy significantly enhanced participants’ physiological and mental well-being, reduced stress levels, and improved employee satisfaction (231). In this process, natural language processing (NLP)-driven continuous learning algorithms play a key role. The system dynamically constructs an emotional state evolution map by continuously analyzing the language expressions of the older adults during their travels (such as diaries, voice feedback, and consultation dialogues). When anxiety is detected, the algorithm will link to a database of MT destinations and prioritize recommendations for forest therapy (such as Aomori Prefecture in Japan) or socially intensive nursing homes (such as the Singapore “Senior Activity Centers”). If positive emotions are identified as consistently increasing, the current rehabilitation cycle is appropriately extended to consolidate the intervention’s effectiveness. This “emotion-scenario” adaptive matching mechanism significantly enhances the responsiveness of psychological interventions. Music therapy, aromatherapy, and massage therapy are also widely used in palliative care for terminally ill patients. Jodie F et al., after reviewing multiple databases and conducting a comprehensive assessment, found that these three therapies effectively alleviate pain and anxiety while enhancing happiness and QoL. Among them, music therapy and massage therapy demonstrated particularly notable effects in pain relief and QoL improvement (232). Xiaotangshan Hot Spring in Beijing, a renowned historical health resort in China, is famous for its unique water quality rich in minerals. Its hot spring water is characterized by high heat, fluoride content, sodium bicarbonate-calcium composition, and mild mineralization. It has been widely applied in medical rehabilitation and health management. Xiaotangshan offers various rehabilitation treatments and hydrotherapy programs tailored for different patient groups and sub-health populations, such as aquatic exercise rehabilitation, whirlpool bubble baths, steam therapy, and herbal baths. These therapies not only improve physical health but also help visitors relax, reduce stress, and alleviate anxiety (98). Ben Massoued et al. analyzed the treatment process of a 21-year-old psychiatric patient and found that combining cognitive behavioral therapy (87) with emotion-focused therapy (EFT) effectively alleviated depressive symptoms in individuals at high risk of psychosis, optimizing disease prognosis (233). Furthermore, Liu et al. conducted a systematic review of non-pharmacological interventions for type 2 diabetes and found that TCM-based emotional therapy significantly reduces clinical symptoms in diabetic patients, relieves anxiety, stress, and depression, and enhances overall well-being and mental health (76). In summary, various non-pharmacological interventions demonstrate significant potential in promoting mental health, alleviating disease symptoms, and improving QoL. From horticultural therapy and hydrotherapy to aromatherapy, music therapy, and TCM emotional therapy, these methods not only enhance physical health but also support emotional well-being and psychological resilience, offering personalized health solutions for diverse populations (see Table 4).

**Table 4:** Emotional regulation and psychological resilience as a non-pharmacological intervention in healthcare.

Intervention	Region	Sample	Health Focus	Key findings	Source
HT	China	Dementia patients	Dementia	HT can make dementia patients less agitated, evoke more positive emotions, and help maintain good cognitive function.	Zhao et al. (230)
Balneotherapy, Spa tourism	Spain	Spa tourists in Maresme, Catalonia	Relaxation, recovery, disease prevention, and treatment	The primary motivation for spa tourism is relaxation and recovery (78.36%), followed by disease prevention and treatment. Balneotherapy shows significant potential as a non-pharmacological intervention to promote physical and mental health.	Torres-Pruñonosa et al. (97)
Aromatherapy	China	44 office workers	Stress, anxiety, depression	Aromatherapy positively impacts participants’ emotions, enhances emotional well-being, helps relieve stress, anxiety, and depression, and improves overall physical and mental health.	Liu et al. (231)
Music Therapy, Aromatherapy, Massage Therapy	/	Palliative care for terminally ill patients (based on literature review)	Palliative care	These three therapies improve pain and anxiety in patients, enhance their well-being, and improve QoL.	Jodie et al. (232)
Balneotherapy	Xiaotangshan Hot Spring, Beijing, China	/	Wellness, rehabilitation, healing, and treatment	Balneotherapy helps relax the mind and body, relieve stress, improve anxiety and depression, manage chronic diseases, and support rehabilitative exercise.	Beijing Xiaotangshan Hospital (98)
EFT	British	A 21-year-old psychiatric patient	Depression	EFT significantly improves depressive symptoms and physical functioning in the psychiatric patient.	Ben Massoued et al. (233)
TCM-Emotional Therapy (a NPT)	China	Type 2 diabetes patients	Type 2 diabetes	For patients with diabetic nephropathy, negative emotions are improved, and QoL is enhanced after TCM emotional care. TCM emotional therapy plays a role in improving clinical symptoms, reducing post-void residual urine volume, and alleviating anxiety and depression.	Liu et al. (76)

### 4.5 Physical therapy and rehabilitation in MT

The final aspect to consider is physical therapy and rehabilitation, which represents one of the most distinctive functions of MT compared to regular tourism. Against the backdrop of global population aging, there has been a trend toward younger onset of chronic diseases, making chronic disease management a critical issue in public health. Chronic diseases refer to medical conditions that require long-term management and monitoring, are often difficult to cure, and necessitate ongoing treatment (234). Common chronic diseases include hypertension, diabetes, asthma, depression, osteoporosis, osteoarthritis, and AD. These conditions not only severely impact an individual’s QoL but also place a significant burden on public health systems (1). Chronic disease management is a core component of public health management and a key strategy for slowing the progression of major illnesses (13). Existing statistics reveal that over 80% of individuals aged 65 and above suffer from at least one chronic disease, while 68% have two or more conditions, referred to as multimorbidity (125). These specific pathologies can be fatal individually or, through their combined effects, accelerate mortality risk (115). Consequently, effective chronic disease management has become increasingly urgent. Röhrich, Giordano, and Kohls emphasize that chronic non-healthy states cannot be cured through traditional acute care models but can be alleviated through complementary, health-promoting integrated approaches aimed at symptom relief, functional recovery, and QoL improvement (235). In the context of MT, high-quality physical therapy, natural therapeutic interventions, comprehensive healthcare services, and professional support offer critical solutions for chronic disease management among older adults. These measures not only assist patients in better managing the challenges posed by chronic diseases but also enable personalized health management through multidisciplinary collaboration, thereby enhancing overall health levels and QoL. By integrating advanced medical practices with holistic wellness strategies, MT provides a unique platform for addressing the complex needs of individuals living with chronic conditions, ultimately fostering healthier aging and improved well-being.

For example, Shahzad Khan and Md. Shariful Alam’s research on the current status and potential of MT in Saudi Arabia revealed that the country attracts a large number of tourists for neurological rehabilitation, occupational therapy, and physical therapy due to its advanced healthcare facilities, professional service teams, and cutting-edge rehabilitation technologies, particularly for conditions such as stroke and cerebral palsy-induced neurological disorders (59). This study highlights the significant advantage of MT destinations in attracting specific patient groups through specialized rehabilitation services. Chia-Wen Lee and Ching Li further evaluated the feasibility and appropriateness of the health tourism destination index. Their findings indicated that when selecting an MT destination, individuals prioritize the medical standards, health promotion capabilities, and leisure tourism experiences offered by the destination. Additionally, physical activity and exercise therapy received increased recognition from expert panels, reflecting their growing importance in health management (236). Hussam Abdeljabar Ahmad Mizher et al., based on the PRISMA guidelines, conducted a systematic quality analysis of studies on hypertension and natural therapies retrieved from databases. The results demonstrated that natural therapies not only effectively lower blood pressure but also do not produce significant side effects (17). This provides a new non-pharmacological intervention approach for chronic disease management. Crooks VA et al. conducted semi-structured telephone interviews with Canadian medical tourists who traveled abroad for osteoarthritis surgery. The study found that these tourists generally did not question the necessity of surgery and exhibited a proactive attitude toward surgical interventions. Furthermore, they were willing to actively participate in health-related decision-making processes (237). This study reveals the autonomy and enthusiasm of medical tourists in treatment selection. Ortega-Collazos et al. conducted a study involving 42 older adult patients with osteoarthritis (mean age: 70 years), assessing the effects of peloids (94). The results showed that this therapy significantly alleviated pain, improved functional recovery, and enhanced QoL (95). This indicates the potential value of traditional natural therapies in geriatric chronic disease management. Ali et al. assessed 1,108 medical tourists referred from six countries to the United States for diabetes treatment and care. The study found that short-term diabetes consultations in the context of MT yielded significant outcomes (238). This highlights the efficiency and practicality of MT in chronic disease management. Finally, Liu SJ et al., following the PRISMA evaluation guidelines, analyzed relevant literature in databases and discovered that combining traditional Chinese fitness exercises with medication can significantly increase BMD levels in postmenopausal women (239). This provides important reference data for skeletal health management in older populations. In summary, these studies demonstrate the significant potential of MT in chronic disease management, rehabilitation treatment, and health promotion. From natural therapies to traditional fitness exercises and personalized medical consultations, various interventions offer diverse health management options tailored to different populations (see Table 5).

**Table 5:** Physical therapy and rehabilitation as a non-pharmacological intervention in healthcare (chronic diseases and other conditions).

Intervention	Region	Sample	Health Focus	Key Findings	Source
Healthcare, physical therapy, post-surgical rehabilitation	Saudi Arabia	Medical tourists in Saudi Arabia	Various diseases	Saudi Arabia attracts numerous tourists due to its advanced organ transplant facilities, other medical equipment, highly skilled professionals, and comprehensive healthcare services.	Shahzad Khan and Md. Shariful (59)
Healthcare, sports, natural environment	China	Experts in healthcare, health and tourism sectors, social tourism agencies, and government industrial units	MT destination index	The development of health tourism destinations should focus on medical care, health management, nursing, and recovery. Sightseeing and other leisure activities, along with healthy diets, can improve health.	Lee and Li (236)
Natural therapies (including traditional medicines, herbs, and plants)	/	Studies on natural therapy interventions for hypertension retrieved from databases	Hypertension	Natural therapies are generally safe and well-tolerated for hypertension patients. Most adverse reactions are mild and can be controlled without treatment.	Hussam Abdeljabar Ahmad Mizher and Muhammad Iqbal Hamidullah and Syahrir (17)
MT—cross-border physical therapy	Canada	Canadian medical tourists who traveled abroad for OA treatment	OA	Medical tourists voluntarily and actively decided to undergo surgical interventions for OA treatment and actively cooperated with health management requirements.	(237)
Peloid therapy (94), Mud Baths	Span	42 OA patients with an average age of 70 years	OA	Peloid therapy reduces anxiety, alleviates pain, improves QoL, and enhances physical function in OA patients. Supplementing with RosA can enhance the therapeutic effects of peloid therapy for OA management.	Ortega-Collazos et al. (95)
MT—cross-border physical therapy	America	1,108 medical tourists referred from six countries for diabetes treatment and care in the U. S.	Diabetes	After a single consultation, diabetes symptoms significantly improved. In the short term, MT-based diabetes consultations show significant effectiveness.	Ali et al. (238)
Traditional Chinese Fitness Exercises (Tai Chi, Baduanjin, Wuqinxi, Yijinjing, Liu Zi Jue) Combined with Pharmacological Treatment	\	3,658 postmenopausal women	BMD	Among traditional Chinese fitness exercises, Tai Chi is most effective in improving lumbar spine BMD and Ward’s triangle BMD, while Baduanjin is most effective for femoral neck BMD treatment.	Liu et al. (239)

**Figure 6:**
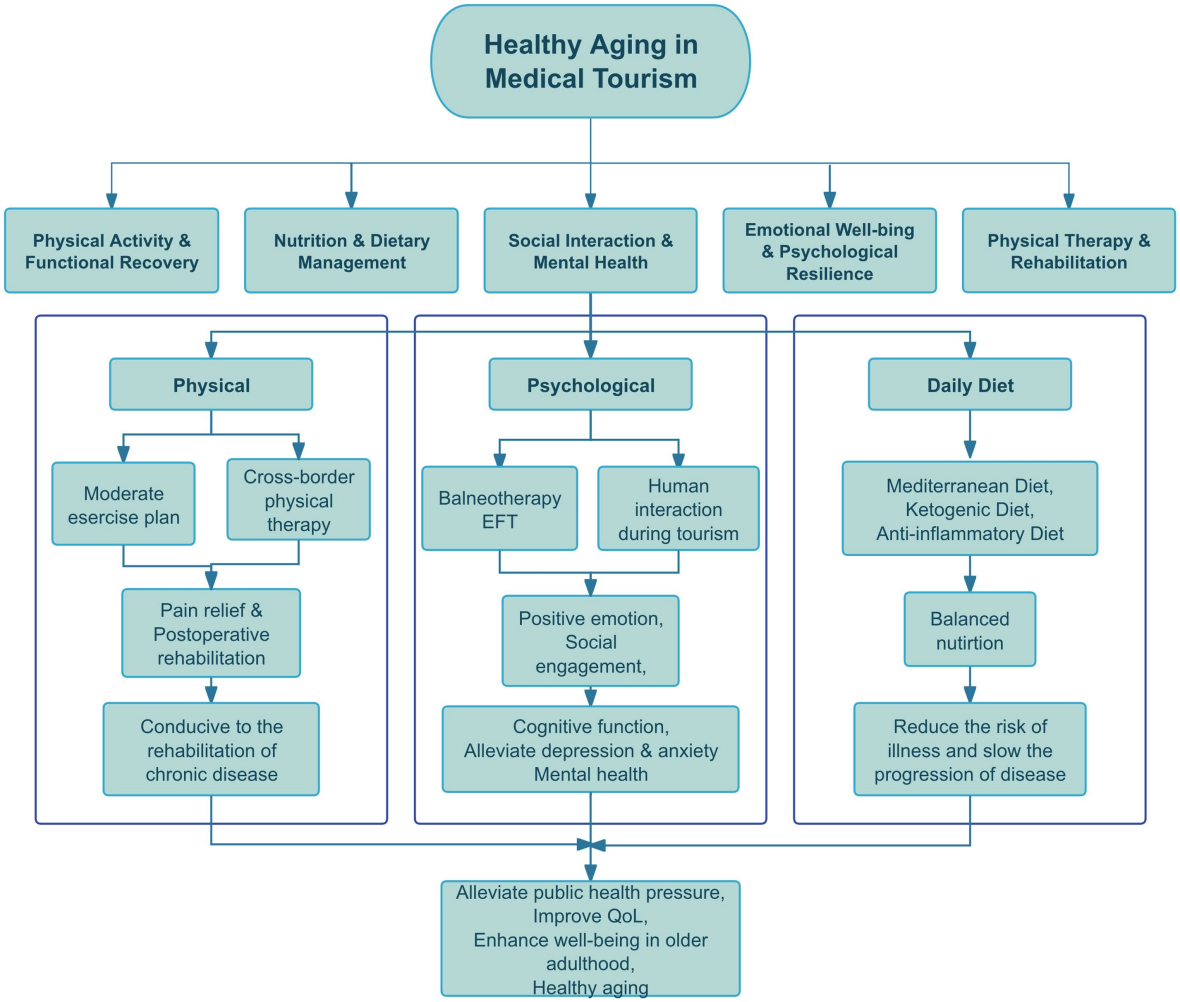
The comprehensive mechanism of proactive health among the older adult patients with chronic conditions in MT. MT supports healthy aging by integrating physical exercise with cross-border healthcare for rehabilitation and chronic disease management, offering mental health interventions such as balneotherapy and psychological counseling to reduce depression and enhance cognition, and providing personalized nutritional plans to optimize dietary health. These combined approaches promote active health management and improve overall well-being among the older adults.

MT serves as a crucial tool for promoting healthy aging by integrating various aspects such as physical activity, nutritional management, social interaction, mental health interventions, physical therapy rehabilitation, and chronic disease management into a comprehensive health management solution for older adults, as shown in Figure 6. Additionally, MT combines personalized medical services and rehabilitation support to meet the diverse and individualized health needs of older adults. Research indicates that during the process of healthy aging, environmental factors and lifestyle choices play a more decisive role compared to genetic factors (125, 128). Non-pharmacological interventions, such as physical exercise, improved nutrition, social participation, and emotional regulation, are particularly emphasized due to their significant cost-effectiveness advantages. These measures are considered among the optimal pathways for optimizing the health of older adults. By addressing both physical and psychological well-being through tailored programs, MT not only enhances the quality of life for older adults but also supports the prevention and management of age-related diseases, ultimately contributing to healthier aging outcomes.

MT offers comprehensive health management services for the older adults by integrating interventions such as exercise rehabilitation, personalized dietary plans, and emotional therapy. These measures not only enhance functional capacity and reduce chronic disease risks but also strengthen social interaction skills and psychological resilience. For example, combined traditional and modern therapies like TCM massage, Tai Chi, and balneotherapy effectively alleviate physical symptoms and significantly improve mental health, thereby enhancing overall QoL (68, 206). Additionally, MT provides telehealth management, post-surgical rehabilitation support, and precision medical testing to ensure continuous health care and personalized attention for older adults. Thus, MT serves not only as a practical health promotion tool but also as an integrated intervention approach that plays a vital role in healthy aging. By combining traditional medicine with modern medical technology, MT offers multidimensional health management solutions and complements conventional healthcare models. Looking ahead, greater integration of MT into public health policies is needed to promote its application in global healthy aging strategies. This will optimize older adults health, enhance well-being and QoL, and provide critical support for achieving society’ s goal of comprehensive healthy aging.

## 5 Limitations and future prospects of current research

This chapter aims to objectively examine the primary limitations and challenges associated with natural non-pharmacological therapies and MT in promoting active health management among older adult patients with chronic diseases and their synergistic role in public health, based on a systematic review of this study and relevant literature. It also proposes forward-looking future research directions based on these findings. This study focuses on the deep integration and efficiency optimization of this field, identifying the following four interrelated key dimensions that require urgent strengthening: first (5.1), the potential of traditional Chinese medicine (as one of the core representatives of natural non-pharmacological therapies) within the MT framework remains significantly underutilized and lacks integration; second (5.2), providing comprehensive MT services that meet the holistic needs of the older population requires breaking down disciplinary barriers and strengthening cross-disciplinary collaboration mechanisms; Third (5.3), the interdisciplinary empirical research foundation supporting MT practices aimed at achieving healthy aging remains weak, particularly lacking scientific quantitative assessments of long-term effects; finally (5.4), in response to the dynamic and individualized needs of older adults chronic disease management, precision health management driven by intelligent algorithms represents an important direction for technological evolution. These limitations and prospects are not isolated but collectively outline the core pathways for future development in this field: from deepening the integrated application of specific natural non-pharmacological therapies (such as traditional Chinese medicine) in MT (5.1), to building a comprehensive service system through multidisciplinary collaboration (5.2), to strengthening evidence-based research to validate intervention effects (5.3), and ultimately moving toward data-driven intelligent health management (5.4). In-depth exploration of these issues not only helps address current research gaps but also provides practical theoretical guidance and implementation pathways for optimizing the synergistic model of MT and natural non-pharmacological therapies, enhancing health management for older adults with chronic health conditions, and advancing the implementation of healthy aging strategies.

### 5.1 Insufficient application of TCM therapy in MT

TCM is a time-honored holistic medical system with a practice history spanning over two millennia. Rooted in the philosophy of balance and harmony, TCM encompasses diverse therapeutic modalities such as acupuncture, herbal medicine, dietary therapy, qigong, and tuina (240). At its core, TCM emphasizes the concept of “unity between heaven and humanity,” viewing the human body as a microcosm interacting with the natural environment, where health arises from the equilibrium of qi, blood, yin, and yang (241). Additionally, TCM theory posits that kidney essence deficiency is the fundamental cause of aging, vitality loss is a key characteristic of aging, and physical and mental exhaustion represent the manifestations of senescence (242). The theoretical framework of TCM is built upon classical texts such as the *Huangdi Neijing*, *Shennong Bencao Jing*, and *Shanghan Lun*, which established foundational principles including yin-yang theory, wuxing (five phases), the meridian system, and zang-fu (visceral organ) physiology (243). In this system, “qi” is regarded as the fundamental life energy that flows through the body via meridians. When the flow of qi is obstructed or imbalanced, disease occurs (244). To accurately assess a patient’s condition, TCM employs the Eight Principles Differentiation (yin/yang, exterior/interior, cold/heat, deficiency/excess) and the Four Diagnostic Methods (observation, auscultation/olfaction, inquiry, pulse palpation) to develop personalized treatment plans (245). In modern healthcare, practitioners can integrate contemporary innovations with TCM’s gerontological knowledge to offer comprehensive and individualized care. This approach focuses not only on physical well-being but also on emotional and spiritual health, enhancing overall QoL (246). As such, TCM serves as an integrative medical system with significant value in promoting healthy aging and improving overall QoL.

TCM treatment methods encompass a variety of therapies that integrate traditional wisdom with modern applications, addressing physical, emotional, and spiritual health. Below is a summary of the key approaches:

#### 5.1.1 Acupuncture and tuina

Acupuncture and tuina are hallmark non-pharmacological therapies in TCM. They stimulate specific acupoints along meridians to regulate the flow of qi and blood, achieving therapeutic effects (247). Modern research highlights acupuncture’s efficacy in pain management, stress reduction, and promoting relaxation by stimulating nerves, muscles, and connective tissues. Tuina, as a form of manual therapy, enhances blood circulation, alleviates muscle tension, and is widely used for musculoskeletal disorders, chronic pain, and sub-health conditions.

#### 5.1.2 Herbal therapy

Herbal therapy forms the core of TCM, utilizing natural herbs combined into formulas to tonify qi and blood, remove dampness and toxins, and harmonize organ functions. Common herbs like ginseng, astragalus, and angelica root have been proven effective in boosting immunity, improving digestive function, and alleviating insomnia (248). These herbs are not only integral to TCM but are increasingly incorporated into modern integrative medicine for treating chronic diseases and functional disorders.

#### 5.1.3 Dietary therapy

TCM dietary therapy emphasizes the concept of “food as medicine,” advocating balanced nutrition to nurture the body and prevent chronic illnesses. For instance, integrating herbal soups or Mediterranean-style diets can effectively address metabolic syndrome and cardiovascular issues. Modern nutritional science aligns with TCM principles, emphasizing whole-food intake and personalized meal planning to optimize health (249).

#### 5.1.4 Moxibustion and cupping

Moxibustion and cupping are common external therapies in TCM. Moxibustion uses heat to improve circulation, expel cold and dampness, and is often applied for rheumatic diseases, dysmenorrhea, and gastrointestinal disorders (250), frequently combined with acupuncture for enhanced results. Cupping employs negative pressure to stimulate blood flow and relieve muscle pain, gaining popularity among athletes and those seeking alternative treatments. Its applications extend to alleviating chronic fatigue, boosting immune function, and aiding post-surgical recovery (251).

#### 5.1.5 Qigong and Tai Chi

Qigong and Tai Chi exemplify TCM’ s movement-based therapies. Through controlled breathing and gentle movements, they enhance vitality, cardiovascular health, balance, and emotional regulation (193). Tai Chi, with its low-impact nature, suits all age groups and fitness levels, improving posture, flexibility, and stress management. Qigong activates the body’s self-healing mechanisms, fostering self-awareness and empowerment, making it a vital tool for holistic well-being (252, 253).

#### 5.1.6 Music therapy

Rooted in the five-element theory, TCM music therapy links musical tones to the functions of internal organs, maintaining internal harmony. It features five basic tones—Jue (254), Zhi (fire), Gong (255), Shang (metal), and Yu (water)—each corresponding to different organ systems (256, 257). These tones regulate emotions, reduce anxiety, and promote overall mind–body balance through auditory stimulation. Together, these therapies reflect TCM’s comprehensive approach to health, blending ancient practices with contemporary insights to address diverse health needs and enhance QoL.

As global health paradigms shift from disease treatment to prevention and holistic health management, TCM has increasingly gained recognition internationally due to its focus on integrative care, personalized treatment, and minimal side effects. The WHO has included TCM in its Global Strategy on Traditional Medicine, and many researches have adopted it as a key component of complementary and alternative medicine (258–260). Despite its proven efficacy in modulating immune function, anti-inflammatory effects, improving blood circulation, and alleviating chronic pain (261, 262), the utilization of TCM within MT remains limited. Current MT offerings predominantly emphasize surgical procedures and rehabilitation therapies, often neglecting holistic health management approaches (263). Consequently, TCM’s comprehensive philosophy has yet to be fully integrated into MT frameworks. Notably, Western mind–body therapies such as yoga and mindfulness meditation are widely adopted in MT because they align more closely with Western medical standards (264–266). In contrast, despite its unique advantages, TCM lacks standardized protocols that could systematically integrate its practices into MT programs. For instance, the combination of acupuncture, balneotherapy (hot spring therapy), and dietary therapy remains underdeveloped, limiting TCM’s potential in the global health tourism market. Furthermore, research on healthy aging tends to overlook TCM, focusing instead on Western pharmacology, exercise interventions, and psychological therapies while ignoring TCM’s distinct contributions in emotional therapy, qigong-based wellness practices, and dietary interventions. TCM’s emotional therapy holds significant potential for preventing geriatric depression, and Tai Chi practice has been shown to improve balance and cardiovascular health among older adults (193). These methods are cost-effective and scalable but remain in the early stages of investigation. Therefore, future research should prioritize exploring the application of TCM in MT by developing standardized TCM-based health management solutions, such as integrated models combining acupuncture, balneotherapy, and dietary therapy. Additionally, integrating TCM principles into frameworks for healthy aging research is essential to better understand its role in preventing and managing age-related diseases. To enhance the appeal of MT destinations and meet growing global health demands, interdisciplinary collaboration between medicine and tourism management is necessary. This includes designing and promoting health tourism products that incorporate TCM treatments and wellness philosophies. Such efforts can not only address gaps in current MT offerings but also provide comprehensive, culturally enriched health solutions tailored to an aging global population.

### 5.2 Insufficient interdisciplinary collaboration in MT to provide comprehensive services

MT should extend beyond medical treatment to encompass multi-dimensional health support, including mental health, dietary management, and exercise rehabilitation, in order to meet the comprehensive health management needs of older adults. As global population aging intensifies, the number of older adult tourists is rising, along with their demand for integrated medical services. Older adult tourists require not only chronic disease management and post-surgical rehabilitation but also scientifically designed exercise programs to promote physical activity and recovery, as well as dietary therapy to maintain health and gently improve their physical condition. Furthermore, psychological interventions such as music therapy and mindfulness meditation can effectively help older adults prevent depression, anxiety, age-related social stigma, and cognitive impairments, thereby enhancing their QoL (193, 266). However, current MT programs predominantly focus on single-service offerings, such as cross-border cosmetic treatments, post-surgical rehabilitation, and reproductive medicine, lacking an integrative, interdisciplinary health management framework (267). Despite the need for comprehensive interventions in healthy aging, existing MT projects remain centered on single-disease management, failing to adequately address lifestyle interventions, dietary therapy, and long-term health monitoring (263). This limitation makes it difficult for tourists aged 65 and above to access systematic health promotion services that fully satisfy their diverse needs for healthy aging. To better meet the needs of older adult tourists, MT destinations should provide integrated health services by adopting a “three-dimensional holistic health management” model that combines medical care, rehabilitation, mental health, dietary management, and exercise management into a unified system. Specifically, MT destinations are encouraged to strengthen interdisciplinary collaboration among doctors, nutritionists, exercise rehabilitation specialists, and psychotherapists to tailor personalized health management plans for older adult tourists, ensuring they receive comprehensive support throughout their MT journey. Additionally, destinations should integrate multidisciplinary resources to establish comprehensive intervention centers for healthy aging, offering an integrated service model combining medical care, rehabilitation, mental health, dietary management, and exercise management to enhance the overall health experience of tourists aged 65 and above. Technology-enabled solutions are critical for achieving precise health management. MT destinations can leverage wearable devices to monitor the real-time health status of older adult tourists and combine telemedicine technologies to provide continuous health management support. These technological tools not only improve the efficiency and accuracy of health management but also offer personalized health guidance, further enhancing older adult tourists’ trust and satisfaction with MT services (264). MT destinations should transition from a single-disease management model to a systemic health promotion model by integrating multidisciplinary resources, strengthening interdisciplinary collaboration, and incorporating advanced technology. Crucially, interdisciplinary collaboration requires technology to connect data. Continuous learning algorithms can build a dynamic knowledge integration platform: through incremental learning mechanisms, they integrate doctors’ diagnoses, nutritionists’ dietary plans, rehabilitation therapists’ exercise prescriptions, and psychological assessment data to generate cross-domain health profiles while protecting privacy. For example, when a traditional Chinese medicine practitioner inputs a patient’s diagnosis of “liver qi stagnation and spleen deficiency,” the algorithm automatically links nutritionists’ low-fat, high-fiber meal plans, psychologists’ music therapy programs, and physical therapists’ tai chi training plans, forming a personalized MT service package guided by the holistic view of traditional Chinese medicine. By doing so, they can create a comprehensive health service system tailored to the needs of older adult tourists, providing robust support for achieving the goal of healthy aging.

### 5.3 Insufficient interdisciplinary empirical research on MT in healthy aging

Current research on how MT promotes healthy aging primarily focuses on theoretical exploration, with a notable lack of interdisciplinary empirical studies, particularly regarding the long-term quantifiable impacts of MT on aging populations. Existing studies predominantly employ qualitative methods, such as surveys or in-depth interviews, to examine the travel experiences of older adult tourists and their potential health benefits. For instance, Miyashita et al. (268) conducted a survey of Japanese retirees who traveled to Thailand for chronic disease treatment or post-surgical rehabilitation, revealing improvements in their health status following these interventions. Peng et al. (136) extended this research by applying transformative service research, highlighting how MT enhances the well-being of vulnerable groups, such as middle-aged and older adults, through non-pharmacological interventions. While these studies provide initial insights into the value of MT, their limitations are significant: they rely heavily on qualitative approaches, lacking robust quantitative analyses that could comprehensively evaluate MT’s specific impact on healthy aging. Future research should integrate medical research methodologies, utilizing rigorous empirical methods such as randomized controlled trials and case–control studies, to scientifically assess both the short-and long-term effects of MT on aging populations. High-quality evidence-based research would offer more compelling clinical evidence to validate whether MT effectively promotes physical, psychological, and social health among older adults. Additionally, such studies should compare the efficacy of MT with other health management approaches to clarify its unique advantages and appropriate applications. A notable challenge lies in the measurement of healthy aging, a multidimensional concept encompassing physiological health, cognitive function, mental state, and social well-being. There is currently no consensus on standardized metrics for assessing healthy aging. For example, Lara et al. (269) defined objective indicators of healthy aging as cognitive function, physical capacity, physiological function, immune function, and endocrine function, emphasizing the importance of biomarkers. In contrast, Sanchez-Niubo et al. (204) developed the ATHLOS scale, which integrates assessments of cognition, vitality, psychological symptoms, sensory function, mobility, and activities of daily living. This diversity in measurement systems reflects the complexity of healthy aging but also complicates comparability and consistency across studies. To enhance the scientific rigor and accuracy of future research, reliance solely on contributions from tourism scholars is insufficient. Interdisciplinary collaboration involving medicine, psychology, sociology, gerontology, public health, and tourism management is essential. Such collaborations can optimize measurement methods for healthy aging and deepen understanding of the practical health benefits of MT as a non-pharmacological intervention. For example, integrating biomarker detection techniques from medical research with mental health assessment tools from psychology could provide more precise evaluations of MT’s impact on older adult health. Incorporating perspectives from sociology and gerontology would further illuminate MT’s broader implications for social well-being and QoL. Future research should prioritize three key directions: 1. adopting more scientifically rigorous methodologies, such as RCTs and case–control studies, to quantify MT’s health benefits; 2. fostering interdisciplinary cooperation to develop unified and comprehensive frameworks for measuring healthy aging; and 3. exploring the potential applications of MT within global healthy aging policies and practices, providing robust theoretical foundations and scientific support for policy formulation and industry implementation. Through these efforts, MT can be better positioned as an effective tool for promoting comprehensive and sustainable healthy aging.

### 5.4 Dynamic health management driven by intelligent algorithms

Current MT services have significant limitations in dynamically responding to the needs of older adult patients with chronic conditions. However, intelligent algorithms are reshaping the paradigm of health management by constructing a “perception-decision-optimization” closed-loop system. This system first integrates multi-source data to create a dynamic health profile, combining four dimensions: environmental (climate and air quality of the health resort), behavioral (adherence to non-pharmacological therapies), physiological (metrics from wearable devices), and feedback (patient subjective evaluations). Gradient masking technology is employed to achieve incremental data fusion, effectively avoiding the catastrophic forgetting issue of traditional models. Its technical framework has been validated through the DeepSeek-R1 health profile model. Based on this, the algorithm generates personalized MT path planning based on health profiles and efficacy predictions, dynamically optimizing health resort switching strategies through a multi-armed bandit model: in the initial stage, it recommends Japanese Hakone hot spring therapy to alleviate joint pain; in the advanced stage, it switches to Thai Chiang Mai to regulate metabolic syndrome; and in the consolidation stage, it selects Chinese Hainan to prevent cognitive decline. This intelligent recommendation mechanism has been integrated into the Ctrip MT system. Meanwhile, the risk warning system constructed by the temporal convolutional network can identify three high-risk scenarios seven days in advance—hypoxia reactions in high-altitude areas, blood sugar fluctuations in tropical dietary environments, and fall risks from high-intensity activities—and push customized intervention plans. Its technical prototype has achieved significant results in the Ping An Health Insurance system.

In the field of traditional Chinese medicine, algorithms have broken through the bottleneck of traditional experience inheritance. Through deep learning, they analyze the diagnostic rules of renowned physicians, associate tongue pattern maps and pulse data with syndromes such as “liver and kidney yin deficiency,” and construct acupuncture prescription optimization models. This new interdisciplinary collaboration framework not only enables intelligent analysis of medical data but also promotes the deep integration of traditional medicine and modern algorithms. From data collection to dynamic planning, from risk warning to TCM standardization, intelligent algorithms are building a smart health management ecosystem covering the entire life cycle through multi-dimensional technological collaboration.

While medical tourism offers promising opportunities for health promotion and access to alternative treatments, it also presents several critical challenges. These include regulatory disparities across countries, limited oversight of treatment quality and safety, and the risk of exploitation of vulnerable patients. Additionally, issues such as continuity of care—where patients return home after treatment without follow-up—pose significant risks to long-term health outcomes. There are also ethical concerns related to healthcare equity, as MT often benefits wealthier individuals while potentially diverting medical resources away from local populations in destination countries.

## 6 Conclusion

This study underscores the potential of MT as a proactive strategy for promoting healthy aging. By integrating natural non-pharmaceutical therapies with a dynamic health portrait framework, MT can support personalized and preventive care for older adults. However, the current conceptual model lacks empirical validation. Future research should incorporate AI-powered continual learning systems and longitudinal tracking to construct adaptive, multimodal health portraits that reflect real-time environmental, behavioral, physiological, and feedback data. This approach will not only improve the accuracy and relevance of health interventions but also enhance equity and quality of life in aging populations, offering scalable solutions for global public health challenges.

## References

[1] Kaplan A Emre B. Evaluation of health literacy level and personal factors in disease self-management of emergency department patients with chronic diseases. Int Emerg Nurs. (2024) 77:101523. doi: 10.1016/j.ienj.2024.101523, PMID: 39378713

[2] Dave U Lewis EG Patel JH Godbole N. Private health insurance in the United States and Sweden: a comparative review. Health Sci Rep. (2024) 7:e1979. doi: 10.1002/hsr2.1979, PMID: 38495896 PMC10940498

[3] Johnston-Webber C Bencomo-Bermudez I Wharton G van Kessel R Barone S Muntó FB. A conceptual framework to assess the health, socioeconomic and environmental burden of chronic kidney disease. Health Policy. (2025) 152:105244. doi: 10.1016/j.healthpol.2024.105244 39827831

[4] World Health Organization (2022). World Health Statistics 2022. Available online at: https://www.who.int/news/item/20-05-2022-world-health-statistics-2022

[5] Yiru W Siyao W Peng G Qiaodan X Lixia L. Survey on knowledge related to Alzheimer’s disease. Armed Forces Med. (2024) 35:1071–6. doi: 10.14010/j.cnki.wjyx.2024.12.015

[6] Turner L. ‘First world health Care at Third World Prices’: globalization, bioethics and medical tourism. Bio Soc. (2007) 2:303–25. doi: 10.1017/S1745855207005765

[7] Christine NBACY. Therapeutic landscapes and postcolonial theory: a theoretical approach to medical tourism. Soc Sci Med. (2012) 74:783–7. doi: 10.1016/j.socscimed.2011.11.016 22305946

[8] Heung VCS Kucukusta D Song H. A conceptual model of medical tourism: implications for future research. J Travel Tourism Mark. (2010) 27:236–51. doi: 10.1080/10548401003744677

[9] Majeed S Lu C Tariq J. The journey from an allopathic to natural treatment approach: a scoping review of medical tourism and health systems. Eur J Integr Med. (2017) 16:22–32. doi: 10.1016/j.eujim.2017.10.001

[10] World Health Organization. (2024). Aging. Retrieved 1. Available online at: https://www.who.int/health-topics/ageing#tab=tab_1

[11] Chen C Tian Y Ni L Xu Q Hu Y Bin P. The influence of social participation and depressive symptoms on cognition among middle-aged and older adults. Heliyon. (2024) 10:e24110. doi: 10.1016/j.heliyon.2024.e24110 38293386 PMC10825423

[12] Xu J Wang J Wimo A Fratiglioni L Qiu C. The economic burden of dementia in China, 1990–2030: implications for health policy. Bull World Health Organ. (2016) 95:18–26. doi: 10.2471/BLT.15.167726, PMID: 28053361 PMC5180346

[13] Yang M. A community proactive health management model for family doctors in Shandong, China. Aust J Prim Health. (2024) 30:Article PY24030. doi: 10.1071/PY24030, PMID: 38976639

[14] Ooi SL McLean L Cheon S P. Naturopathy in Australia: where are we now? Where are we heading? Complement Ther Clin Pract. (2018) 33:27–35. doi: 10.1016/j.ctcp.2018.07.009 30396623

[15] American Association of Naturopathic Physicians. House of Delegates Position Paper on the definition of naturopathic medicine. Available online at: https://cdn.ymaws.com/naturopathic.org/resource/resmgr/documents/governance_docs/definition_naturopathic_medi.pdf

[16] Ooi SL Rae J Cheon S. Implementation of evidence-based practice: a naturopath perspective. Complement Ther Clin Pract. (2016) 22:24–8. doi: 10.1016/j.ctcp.2015.11.004 26850801

[17] Mizher HAA Hamidullah MI Syahrir Z. Natural remedies for hypertension: a systematic review. Pharmacol Res Nat Prod. (2025) 6:100145. doi: 10.1016/j.prenap.2025.100145

[18] de Vries S van Dillen SM Groenewegen PP Spreeuwenberg P. Streetscape greenery and health: stress, social cohesion and physical activity as mediators. Soc Sci Med. (2013) 94:26–33. doi: 10.1016/j.socscimed.2013.06.030, PMID: 23931942

[19] Ishak SA Hussein H Jamaludin AA. Neighbourhood parks as a potential stress reliever: review on literature. Open House Int. (2018) 43:52–64. doi: 10.1108/OHI-04-2018-B0007

[20] Markevych I Schoierer J Hartig T Chudnovsky A Hystad P Dzhambov AM. Exploring pathways linking greenspace to health: theoretical and methodological guidance. Environ Res. (2017) 158:301–17. doi: 10.1016/j.envres.2017.06.028, PMID: 28672128

[21] Kaplan R Kaplan S. The experience of nature: A psychological perspective. New York: Cambridge University Press (1989).

[22] Ulrich RS. Stress recovery during exposure to natural and urban environments. J Environ Psychol. (1993) 36:729–42. doi: 10.1016/S0272-4944(05)80184-7

[23] Yang Y Ren C Zhang Y Wu X. Ginseng: an nonnegligible natural remedy for healthy aging. Aging Dis. (2017) 8:708–20. doi: 10.14336/AD.2017.0707, PMID: 29344412 PMC5758347

[24] Tolo C Lubogo P Yadesa T. *Catha edulis* (Vahl) Endl., a potential natural remedy for type 2 diabetes mellitus or a complicating factor: a narrative review. Nat Prod Commun. (2024) 19:1934578X241302014. doi: 10.1177/1934578X241302014

[25] Yimer E Tuem K Karim A Ur-Rehman N Anwar F. Nigella sativa L. (black cumin): a promising natural remedy for wide range of illnesses. Evid Based Complement Alternat Med. (2019) 2019:Article 1528635. doi: 10.1155/2019/1528635, PMID: 31214267 PMC6535880

[26] Pohl F Kong Thoo Lin P. The potential use of plant natural products and plant extracts with antioxidant properties for the prevention/treatment of neurodegenerative diseases: in vitro, in vivo and clinical trials. Molecules. (2018) 23:3283. doi: 10.3390/molecules23123283, PMID: 30544977 PMC6321248

[27] Ayaz M Ullah F Sadiq A Kim MO Ali T. Natural products-based drugs: potential therapeutics against Alzheimer’s disease and other neurological disorders. Front Pharmacol. (2019) 10:1417. doi: 10.3389/fphar.2019.01417 31849668 PMC6889855

[28] The Business Research Company. (2022). Medical tourism global market report 2022: By type, by healthcare service, by service providerss. London, United Kingdom. Available online at: https://www.thebusinessresearchcompany.com/report/medical-tourism-global-market-report

[29] Erkanli E Kilic H Ozturen A. Sustainable recovery in health tourism: managerial insights from a Mediterranean destination during the COVID-19 pandemic. Sustainability. (2024) 16:8171. doi: 10.3390/su16188171, PMID: 40881048

[30] Carrera PM Bridges JF. Globalization and healthcare: understanding health and medical tourism. Expert Rev Pharmacoecon Outcomes Res. (2006) 6:447–54. doi: 10.1586/14737167.6.4.447, PMID: 20528514

[31] Jiang L Huazhang W Yang S. Diversified demand for health tourism matters: from a perspective of the intra-industry trade. Soc Sci Med. (2022) 293:114630. doi: 10.1016/j.socscimed.2021.114630, PMID: 34954675

[32] Ormond M Sulianti D. More than medical tourism: lessons from Indonesia and Malaysia on south–south intra-regional medical travel. Curr Issues Tour. (2017) 20:94–110. doi: 10.1080/13683500.2014.937324

[33] de la Hoz-Correa A Muñoz-Leiva F Bakucz M. Past themes and future trends in medical tourism research: a co-word analysis. Tour Manag. (2018) 65:200–11. doi: 10.1016/j.tourman.2017.10.001

[34] Hayati R Ansarizadeh M Yousefi M Moein H Alinejad N Jasour A. Effects of medical tourism on hospital waste as an important criteria of the green hospital. Case Stud Chem Environ Eng. (2025) 11:101051. doi: 10.1016/j.cscee.2024.101051

[35] Borg EA Ljungbo K. International market-oriented strategies for medical tourism destinations. Int J Market Res. (2018) 60:621–34. doi: 10.1177/1470785318770134

[36] Suess C Baloglu S James AB. Perceived impacts of medical tourism development on community wellbeing. Tour Manag. (2018) 69:232–45. doi: 10.1016/j.tourman.2018.06.006

[37] Hadian M Jabbari A Mousavi SH Sheikhbardsiri H. Medical tourism development: a systematic review of economic aspects. Int J Healthc Manag. (2021) 14:576–82. doi: 10.1080/20479700.2019.1677977

[38] Turner LG. Quality in health care and globalization of health services: accreditation and regulatory oversight of medical tourism companies. Int J Qual Health Care. (2011) 23:1–7. doi: 10.1093/intqhc/mzq078, PMID: 21148210

[39] Wang R Geng S. Achieving sustainable medical tourism: unpacking privacy concerns through a tripartite game theoretic lens. Front Public Health. (2024) 12:1347231. doi: 10.3389/fpubh.2024.1347231, PMID: 38655509 PMC11037244

[40] Tang K Yao J Wang R Ruan W Chen X Yang L. Factors influencing proactive health behaviors in pre-frailty older adults: a qualitative study based on theory of planned behavior. Geriatr Nurs. (2024) 60:671–6. doi: 10.1016/j.gerinurse.2024.10.029 39522436

[41] He J Wang T. The community proactive health management model based on the grounded theory: the case of Beijing, China. Heliyon. (2023) 9. doi: 10.1016/j.heliyon.2023.e14992, PMID: 37035378 PMC10069935

[42] Haleem A Javaid M Singh RP Suman R. Medical 4.0 technologies for healthcare: features, capabilities, and applications. Internet Things Cyber-Phys Syst. (2022) 2:12–30. doi: 10.1016/j.iotcps.2022.04.001

[43] Hong YA. Medical tourism and telemedicine: a new frontier of an old business. J Med Internet Res. (2016) 18:e115. doi: 10.2196/jmir.5432, PMID: 27215230 PMC4895094

[44] Jones CA Keith LG. Medical tourism and reproductive outsourcing: the dawning of a new paradigm for healthcare. Int J Fertil Womens Med. (2006) 51:251–5. doi: 10.1093/humupd.dml044 17566566

[45] Lunt N Horsfall D Hanefeld J. Medical tourism: a snapshot of evidence on treatment abroad. Maturitas. (2016) 88:37–44. doi: 10.1016/j.maturitas.2016.03.001, PMID: 27105695

[46] Rydback M Hyder AS. Customization in medical tourism in the Philippines. Int J Pharm Healthc Mark. (2018) 12:486–500. doi: 10.1108/IJPHM-07-2017-0035

[47] Tyan I Guevara-Plaza A Yagüe MI. The benefits of blockchain technology for medical tourism. Sustainability. (2021) 13:12448. doi: 10.3390/su132212448

[48] Wong AKF Li P Pan Y Xu J Jiacheng D. Investigating resident perceptions of medical tourism in Hainan: an integrative analytical approach. Tour Manage Persp. (2024) 54:101305. doi: 10.1016/j.tmp.2024.101305

[49] John C. Medical tourism: sea, sun, sand and surgery. Tour Manag. (2006) 27:1093–100. doi: 10.1016/j.tourman.2005.11.005

[50] Kim S Arcodia C Kim I. Critical success factors of medical tourism: the case of South Korea. Int J Environ Res Public Health. (2019) 16:4964. doi: 10.3390/ijerph16244964, PMID: 31817698 PMC6950601

[51] Kim K Seo B. Developmental strategies of the promotion policies in medical tourism industry in South Korea: a 10-year study (2009-2018). Iran J Public Health. (2019) 48:1607–16. doi: 10.18502/IJPH.V48I9.3019 31700816 PMC6825657

[52] Thomas C. The main paths of medical tourism: from transplantation to beautification. Tour Manag. (2014) 45:49–58. doi: 10.1016/j.tourman.2014.03.016

[53] Chen K-H Chang F-H Wu C. Investigating the wellness tourism factors in hot spring hotel customer service. Int J Contemp Hosp Manag. (2013) 25:1092–114. doi: 10.1108/IJCHM-06-2012-0086

[54] Chen Q. Neurobiological and anti-aging benefits of yoga: a comprehensive review of recent advances in non-pharmacological therapy. Exp Gerontol. (2024) 196:112550. doi: 10.1016/j.exger.2024.112550, PMID: 39173784

[55] Zheng D Wen J Kozak M Phau I Hou H Wei W. Vulnerable populations with psychological disorders in tourism: methodological challenges and recommended solutions for empirical research. Tour Manag. (2023) 98:104760. doi: 10.1016/j.tourman.2023.104760

[56] Wen J Wang CC Goh E Su Z Tianyu Y. Traditional Chinese medicine as a tourism recovery drawcard to boost China's inbound tourism after COVID-19. Asia Pac J Mark Logist. (2021) 34:385–400. doi: 10.1108/APJML-10-2020-0732

[57] Pilkington M. The relation between tokens and blockchain networks: the case of medical tourism in the Republic of Moldova. J British Blockch Assoc. (2020) 4:1–11. doi: 10.31585/jbba-4-1-(2)2021

[58] Ramos V Untong A. Spa tourism In: Jafari J Xiao H, (Eds.), Encyclopedia of tourism. Springer International Publishing (2014). 1–3. doi: 10.1007/978-3-319-01669-6

[59] Khan S Md Shariful A. Kingdom of Saudi Arabia: a potential destination for medical tourism. J Taibah Univ Med Sci. (2014) 9:257–62. doi: 10.1016/j.jtumed.2014.01.007

[60] Vrkljan S Grazio S. Business performance of health Spa tourism providers in relation to the structure of employees in the Republic of Croatia. Acta Clin Croat. (2017) 56:681–8. doi: 10.20471/acc.2017.56.04.15, PMID: 29590723

[61] Wang Z Zhang Y Lu S Tan L Guo W Lown M. Horticultural therapy for general health in the older adults: a systematic review and meta-analysis. PLoS One. (2022) 17:e0263598. doi: 10.1371/journal.pone.0263598 35143551 PMC8830630

[62] Heung VCS Kucukusta D. Wellness tourism in China: resources, development and marketing. Int J Tourism Res. (2013) 15:346–59. doi: 10.1002/jtr.1880

[63] Phang MWL Lew SY Chung I Lim WK-S Lim LW Wong KH. Therapeutic roles of natural remedies in combating hereditary ataxia: a systematic review. Chin Med. (2021) 16:15. doi: 10.1186/s13020-020-00414-x, PMID: 33509239 PMC7841890

[64] Yan Y. Medical tourism in China: traditional medicine serving as an emerging tourism resource In: Luo Y Jiang J Bi D, editors. Tourism product development in China, Asian and European countries. Singapore: Springer Singapore (2020). 189–97. doi: 10.1007/978-981-15-4447-7_12

[65] Peng J Yang X Senhui F Tzung-Cheng H. Exploring the influence of tourists’ happiness on revisit intention in the context of traditional Chinese medicine cultural tourism. Tour Manag. (2023) 94:104647. doi: 10.1016/j.tourman.2022.104647

[66] Amudha R. Pharma tourism: building a healthy and wealthy India. J Hospital Appl Res. (2016) 9:27–9.

[67] Lee JA Pausé CJ. Stigma in practice: barriers to health for fat women. Front Psychol. (2016) 7:2063. doi: 10.3389/fpsyg.2016.02063, PMID: 28090202 PMC5201160

[68] Sanford H Levy MD Abihm F. Chapter 48-chronic fatigue syndrome In: David R, editor. Integrative Medicine. Fourth edn. Philadelphia, PA: Elsevier (2018). 484–492.e482. doi: 10.1016/B978-0-323-35868-2.00048-7

[69] Shin J Lee Y Shin J Lee J Kim H Kim M. Utilization status and satisfaction with medical services in nonresidential foreign medical tourists visiting a Korean medicine hospital. Evid Based Complement Alternat Med. (2018) 2018:Article 6586352. doi: 10.1155/2018/6586352 29853966 PMC5960560

[70] Tharakan YG. Development of a health and wellness Centre at Manipal-an introspection. J Hospital Applic Res. (2012) 7:52–66.

[71] Haun J Nakase-Richardson R Hoffman J Sevigny M Sodders M Hammond F. Provider identified access barriers to delivering acupuncture to persons with traumatic brain injury and chronic pain: a cross-sectional self-report survey. J Integr Complement Med. (2024) 31:145–153. doi: 10.1089/jicm.2024.0486 39558833

[72] Pan S Wang S Xue X Yuan H Li J Liu Y. Multidimensional pain modulation by acupuncture analgesia: the reward effect of acupuncture on pain relief. Evid Based Complement Alternat Med. (2022) 31:145–153., PMID: 36408345 10.1155/2022/3759181 PMC9671730

[73] Valera-Calero J Fernández-de-las-Peñas C Navarro-Santana M Plaza-Manzano G. Efficacy of dry needling and acupuncture in patients with fibromyalgia: a systematic review and meta-analysis. Int J Environ Res Public Health. (2022) 19:9904. doi: 10.3390/ijerph19169904 36011540 PMC9408486

[74] Da X Yue L Li X Chen J Yuan N Chen J. Potential therapeutic effect and methods of traditional Chinese medicine on COVID-19-induced depression: a review. Anat Rec. (2021) 304:2566–78. doi: 10.1002/ar.24758, PMID: 34636498 PMC8652675

[75] Ji W Wu L Pan G Zou X. Effects and safety of non-pharmacological therapies of traditional Chinese medicine for coronary heart disease: an overview of systematic reviews. Evid Based Complement Alternat Med. (2022) 2022:8465269. doi: 10.1155/2022/8465269, PMID: 35345620 PMC8957469

[76] Liu J Yao C Wang Y Zhao J Luo H. Non-drug interventions of traditional Chinese medicine in preventing type 2 diabetes: a review. Chin Med. (2023) 18:151. doi: 10.1186/s13020-023-00854-1, PMID: 37964315 PMC10644617

[77] Yang Y Zhang M Zhao J Song S Hong F Zhang G. Effect of traditional Chinese medicine emotional therapy on post-stroke depression a protocol for systematic review and meta-analysis. Medicine. (2021) 100:e25386. doi: 10.1097/MD.0000000000025386, PMID: 33832127 PMC8036081

[78] Cham T-H Lim Y-M Sia B-C Cheah J-H Ting H. Medical tourism destination image and its relationship with the intention to revisit: a study of Chinese medical tourists in Malaysia. J China Tour Res. (2021) 17:163–91. doi: 10.1080/19388160.2020.1734514

[79] Marina G. Physical therapy as a tourist product of medical tourism in Međimurje county. Obrazovanje za poduzetništvo—E4E: znanstveno stručni časopis o obrazovanju za poduzetništvo. (2019) 9:7–19.

[80] Market.us (2023). Medical Tourism Statistics: Emerged as A Significant Global Industry Where Individuals Travel Across International Borders to Seek Medical Treatment Available online at: https://www.globenewswire.com/en/news-release/2023/09/29/2752064/0/en/Medical-Tourism-Statistics-Emerged-as-A-Significant-Global-Industry-Where-Individuals-Travel-Across-International-Borders-to-Seek-Medical-Treatment.html

[81] Ebrahim AH Ganguli S. A comparative analysis of medical tourism competitiveness of India, Thailand and Singapore. Tourism. (2019) 67:102–15.

[82] Gupta V Poonam D. Medical tourism in India. Clin Lab Med. (2012) 32:321–5. doi: 10.1016/j.cll.2012.04.007 22727009

[83] Liu C Chen X W S. The effect of massage therapy on pain after surgery: a comprehensive meta-analysis. Complement Ther Med. (2022) 71:102892. doi: 10.1016/j.ctim.2022.102892 36309174

[84] Xu H-b Nan-chang S. Traditional Chinese massage therapy for palpitation caused by spine disorders: a case report: traditional Chinese massage therapy for palpitation caused by spine disorders. World J Acupunct-Moxibustion. (2022) 32:253–9. doi: 10.1016/j.wjam.2021.11.002

[85] Zhu Q Fang M Gong L Jiang S Sun W Li J. Gait analysis of patients with knee osteoarthritis before and after Chinese massage treatment. J Tradit Chin Med. (2015) 35:411–6. doi: 10.1016/S0254-6272(15)30117-5 26427110

[86] Godlewska A Mazurek-Kusiak A Soroka A. Push and pull factors influencing the choice of a health resort by polish treatment-seekers. BMC Public Health. (2023) 23:2192. doi: 10.1186/s12889-023-17086-5, PMID: 37940893 PMC10631082

[87] Fitzpatrick KK Darcy A Vierhile M. Delivering cognitive behavior therapy to young adults with symptoms of depression and anxiety using a fully automated conversational agent (Woebot): a randomized controlled trial. JMIR Ment Health. (2017) 4:e19. doi: 10.2196/mental.7785, PMID: 28588005 PMC5478797

[88] Lea J. Retreating to nature: rethinking ‘therapeutic landscapes’. Area. (2008) 40:90–8. doi: 10.1111/j.1475-4762.2008.00789.x

[89] Li Y Li W Liu Y. Remedies from nature: exploring the moderating mechanisms of natural landscape features on emotions and perceived restoration in urban parks. Front Psychol. (2024) 15:1502240. doi: 10.3389/fpsyg.2024.1502240, PMID: 39845549 PMC11750842

[90] Smith M. Holistic holidays: tourism and the reconciliation of body, mind and spirit. Tour Recreat Res. (2003) 28:103–8. doi: 10.1080/02508281.2003.11081392

[91] Smith M Kelly C. Holistic tourism: journeys of the self? Tour Recreation Res. (2006) 31:15–24. doi: 10.1080/02508281.2006.11081243

[92] El-Arabi AM. Natural radioactivity in sand used in thermal therapy at the Red Sea coast. J Environ Radioact. (2005) 81:11–9. doi: 10.1016/j.jenvrad.2004.11.002, PMID: 15748657

[93] Kulisch A Mándó Z Sándor E Lengyel Z Illés A Kósa J. Evaluation of the effects of Lake Heviz sulfur thermal water on skin microbiome in plaque psoriasis: an open label, pilot study. Int J Biometeorol. (2023) 67:661–73. doi: 10.1007/s00484-023-02443-1, PMID: 36864227

[94] Vasil'chenko VF Badalov NG Derkacheva LN. The natural resources of the Expedition Bay as a basis for the creation of the health resort Centre at the coast of the Peter the Great Bay, the sea of Japan. Vopr Kurortol Fizioter Lech Fiz Kult. (2014):53–60. 25314771

[95] Ortega-Collazos E Hinchado MD Otero E López-Jurado CF Gálvez I Legido JL. Balneotherapy (mud-bath therapy) with a peloid enriched with rosmarinic acid enhances clinical outcomes and innate immune benefits in elderly patients with osteoarthritis: a pilot study. Appl Sci. (2024) 14:12017. doi: 10.3390/app142412017

[96] Szabó Z Matlovieova K Molnar E Bujdoso Z Hojcska A. Territorial inequalities of medicinal waters, as natural healing factors, in Hungary. Acta Polytech Hung. (2023) 20:13–31. doi: 10.12700/APH.20.10.2023.10.2

[97] Torres-Pruñonosa J Raya J Crespo-Sogas P Mur-Gimeno E. The economic and social value of spa tourism: the case of balneotherapy in Maresme, Spain. PLoS One. (2022) 17:e0262428. doi: 10.1371/journal.pone.0262428 35100293 PMC8803250

[98] Beijing Xiaotangshan Hospital (2023) The hot springs here can cure diseases. Beijing China. Available online at: https://www.xtshos.com.cn/Html/News/Articles/725.html

[99] Ferrara E Scaramuzzino M Murmura G D'Addazio G Sinjari B. Emerging evidence on balneotherapy and thermal interventions in post-COVID-19 syndrome: a systematic review. Healthcare. (2025) 13:96. doi: 10.3390/healthcare13020096 39857123 PMC11765063

[100] Bojang KP Manchana V. Nutrition and healthy aging: a review. Curr Nutr Rep. (2023) 12:369–75. doi: 10.1007/s13668-023-00473-0, PMID: 37191867

[101] Franzago M Alessandrelli E Notarangelo S Stuppia L Vitacolonna E. Chrono-nutrition: circadian rhythm and personalized nutrition. Int J Mol Sci. (2023) 24:2571. doi: 10.3390/ijms24032571, PMID: 36768893 PMC9916946

[102] Longo VD Anderson RM. Nutrition, longevity and disease: from molecular mechanisms to interventions. Cell. (2022) 185:1455–70. doi: 10.1016/j.cell.2022.04.002, PMID: 35487190 PMC9089818

[103] Mohajeri MH. Nutrition and aging. Int J Mol Sci. (2023) 24:9265. doi: 10.3390/ijms24119265, PMID: 37298216 PMC10253359

[104] Hu F Wen J Phau I Ying T Aston J Wei W. The role of tourism in healthy aging: an interdisciplinary literature review and conceptual model. J Hosp Tour Manage. (2023) 56:356–66. doi: 10.1016/j.jhtm.2023.07.013

[105] Gross DC Dahringer JC Bramblett P Sun C Spangler HB Lynch DH. The relationship between a Mediterranean diet and frailty in older adults: NHANES 2007-2017. Nutrients. (2025) 17:326. doi: 10.3390/nu17020326, PMID: 39861456 PMC11767853

[106] Mahdavi A Bagherniya M Mirenayat M Atkin S Sahebkar A. Medicinal plants and phytochemicals regulating insulin resistance and glucose homeostasis in type 2 diabetic patients: a clinical review In: Sahebkar A Simental-Mendía LE (Eds.), Pharmacological properties of plant-derived natural products and implications for human health, vol. 1308. Cham, Switzerland: Springer International Publishing (2021). 161–83. doi: 10.1007/978-3-030-56151-2_8 33861444

[107] Meydani M Hasan S. Dietary polyphenols and obesity. Nutrients. (2010) 2:737–51. doi: 10.3390/nu2070737, PMID: 22254051 PMC3257683

[108] Park L Friso S Choi S. Symposium 4: vitamins, infectious and chronic disease during adulthood and aging nutritional influences on epigenetics and age-related disease. Proc Nutr Soc. (2012) 71:75–83. doi: 10.1017/S0029665111003302 22051144

[109] Yavari M Kalupahana N Harris B Ramalingam L Zu Y Kahathuduwa C. Mechanisms linking obesity, insulin resistance, and Alzheimer's disease: effects of polyphenols and omega-3 polyunsaturated fatty acids. Nutrients. (2025) 17:1203. doi: 10.3390/nu17071203 40218960 PMC11990358

[110] Karimian M. Natural remedies for vascular diseases. Plant Biotechnol Persa. (2019) 1:1–3. doi: 10.29252/pbp.1.1.1

[111] Liu L Li G Cui L Cai R Yuan Y Gao Z. The health benefits of fermented fruits and vegetables and their underlying mechanisms. Compr Rev Food Sci Food Saf. (2024) 23:e70072. doi: 10.1111/1541-4337.70072, PMID: 39611544

[112] Ingrassia M Bacarella S Bellia C Columba P Adamo M Altamore L. Circular economy and agritourism: a sustainable behavioral model for tourists and farmers in the post-COVID era. Front Sustain Food Syst. (2023) 7:1174623. doi: 10.3389/fsufs.2023.1174623

[113] Lopez-Moreno M Fresán U Del Coso J Aguilar-Navarro M López M Pena-Fernandez J. The OMNIVEG study: health outcomes of shifting from a traditional to a vegan Mediterranean diet in healthy men. A controlled crossover trial. Nutr Metab Cardiovasc Dis. (2024) 34:2680–9. doi: 10.1016/j.numecd.2024.08.008 39358106

[114] Jo M Kim E Lee J. Virtual reality vs. imagery: comparing approaches in guided meditation. Front Psychol. (2024) 15:1472780. doi: 10.3389/fpsyg.2024.1472780, PMID: 39654934 PMC11626082

[115] Keshavarz M Xie K Schaaf K Bano D Ehninger D. Targeting the “hallmarks of aging” to slow aging and treat age-related disease: fact or fiction? Mol Psychiatry. (2023) 28:242–55. doi: 10.1038/s41380-022-01680-x, PMID: 35840801 PMC9812785

[116] López-Otín C Blasco MA Partridge L Serrano M Kroemer G. The hallmarks of aging. Cell. (2013) 153:1194–217. doi: 10.1016/j.cell.2013.05.039 23746838 PMC3836174

[117] Schmauck-Medina T Molière A Lautrup S Zhang J Chlopicki S Madsen HB. New hallmarks of ageing: a 2022 Copenhagen ageing meeting summary. Aging. (2022) 14:6829–39. doi: 10.18632/aging.204248, PMID: 36040386 PMC9467401

[118] Poewe W Seppi K Tanner CM Halliday GM Brundin P Volkmann J. Parkinson disease. Nat Rev Dis Primers. (2017) 3:17013. doi: 10.1038/nrdp.2017.13 28332488

[119] Talbott EO Malek AM Lacomis D. The epidemiology of amyotrophic lateral sclerosis. Handb Clin Neurol. (2016) 138:225–38. doi: 10.1016/B978-0-12-802973-2.00013-6, PMID: 27637961

[120] Elshaikh U Sheik R Saeed RKM Chivese T Alsayed Hassan D. Barriers and facilitators of older adults for professional mental health help-seeking: a systematic review. BMC Geriatr. (2023) 23:516. doi: 10.1186/s12877-023-04229-x, PMID: 37626290 PMC10463345

[121] Heisel MJ Sinden D Team, O. P. FC53: an initial evaluation of online meaning-centered groups (OMG) to promote psychological well-being and reduce distress in older adults. Int Psychogeriatr. (2024) 36:73–3. doi: 10.1017/S1041610224001753

[122] Ries W Dagmar P. Chronological and biological age. Exp Gerontol. (1984) 19:211–6. doi: 10.1016/0531-5565(84)90041-X 6479256

[123] Kaeberlein M. How healthy is the healthspan concept? Gero Sci. (2018) 40:361–4. doi: 10.1007/s11357-018-0036-9, PMID: 30084059 PMC6136295

[124] World Health Organization (2024). World health statistics 2024. Available online at: https://iris.who.int/bitstream/handle/10665/376869/9789240094703-eng.pdf?sequence=1

[125] Chen Y Wu J Tang Y Li Z Wen Q Sun X. Multimorbidity and combined interventions for patients with coronary heart disease in Chinese population: latent class analysis of a multi-center study. Int J Cardiol. (2022) 368:17–26. doi: 10.1016/j.ijcard.2022.08.022 35963444

[126] Chen X Giles J Yao Y Yip W Meng Q Berkman L. The path to healthy ageing in China: a Peking University-lancet commission. Lancet. (2022) 400:1967–2006. doi: 10.1016/S0140-6736(22)01546-X, PMID: 36423650 PMC9801271

[127] Dewhurst M. Linzer K. Maud M. Sandler C. (2022) Living longer in better health: six shifts needed for healthy aging McKinsey Health Institute. New York, NY, USA: McKinsey Health Institute. Available online at: https://www.mckinsey.com/mhi/our-insights/living-longer-in-better-health-six-shifts-needed-for-healthy-aging

[128] World Health Organization. World report on ageing and health. Geneva, Switzerland: World Health Organization (2015).

[129] Vacheron J. Healthy ageing: evidence that improvement is possible at every age. Europ Geriatric Med. (2016) 7:298–305. doi: 10.1016/j.eurger.2016.04.014

[130] Cox LS Faragher RG. Linking interdisciplinary and multiscale approaches to improve healthspan—a new UK model for collaborative research networks in ageing biology and clinical translation. Lancet Healthy Longev. (2022) 3:e318–20. doi: 10.1016/S2666-7568(22)00095-2 36098307

[131] Owusu-Addo E Ofori-Asenso R Batchelor F Mahtani K Brijnath B. Effective implementation approaches for healthy ageing interventions for older people: a rapid review. Arch Gerontol Geriatr. (2021) 92:104263. doi: 10.1016/j.archger.2020.104263, PMID: 33010790

[132] Di Lorito C Long A Byrne A Harwood RH Gladman JR Schneider S. Exercise interventions for older adults: a systematic review of meta-analyses. J Sport Health Sci. (2021) 10:29–47. doi: 10.1016/j.jshs.2020.06.003 32525097 PMC7858023

[133] Sadaqa M Németh Z Makai A Prémusz V Hock M. Effectiveness of exercise interventions on fall prevention in ambulatory community-dwelling older adults: a systematic review with narrative synthesis. Front Public Health. (2023) 11:1209319. doi: 10.3389/fpubh.2023.1209319, PMID: 37601180 PMC10435089

[134] Institute, M. H. (2023). Aging with purpose: Why meaningful engagement with society. Available online at: https://www.mckinsey.com/mhi/our-insights/aging-with-purpose-why-meaningful-engagement-with-society-matters

[135] Rajaram S Jones J Lee GJ. Plant-based dietary patterns, plant foods, and age-related cognitive decline. Adv Nutr. (2019) 10:S422–s436. doi: 10.1093/advances/nmz081, PMID: 31728502 PMC6855948

[136] Peng J Yang X Poon P Lishan X. Enhancing users' well-being in virtual medical tourism communities: a configurational analysis of users’ interaction characteristics and social support. Technol Soc. (2022) 71:102084. doi: 10.1016/j.techsoc.2022.102084

[137] Hajra V Aggarwal A. Unveiling the antecedents of senior citizens′ behavioural intentions to travel: a mixed-method approach. Tour Hosp Res. (2023) 23:312–31. doi: 10.1177/14673584221085459

[138] Ward A. Segmenting the senior tourism market in Ireland based on travel motivations. J Vacat Mark. (2014) 20:267–77. doi: 10.1177/1356766714525775

[139] Hwang J Lee J. Antecedents and consequences of brand prestige of package tour in the senior tourism industry. Asia Pac J Tourism Res. (2019) 24:679–95. doi: 10.1080/10941665.2019.1623274

[140] Wijaya S Wahyudi W Kusuma CB Sugianto E. Travel motivation of Indonesian seniors in choosing destination overseas. Int J Cult Tourism Hosp Res. (2018) 12:185–97. doi: 10.1108/IJCTHR-09-2017-0095

[141] Goh E Tham A Lai MY. Exploring senior solo travel through a tri-factor healthy ageing framework. Tour Manage Perspect. (2024) 54:101314. doi: 10.1016/j.tmp.2024.101314

[142] DeMicco F. Medical tourism and hospitality bridging healthcare (H2H): disruptive technologies and innovation for patient/guest solutions In: Reference module in social sciences. Amsterdam, The Netherlands: Elsevier (2024)

[143] Lu Z Yu L Fan K Hu T Liu L Li S. Associations between social support and proactive health behaviours among Chinese adolescents: the mediating role of self-efficacy and peer relationships. BMC Public Health. (2024) 24:2548. doi: 10.1186/s12889-024-20070-2, PMID: 39300420 PMC11412034

[144] Huang JS. Health management in USA: originated from uncontrollable growth of medical care expenditure. Zhonghua Yi Xue Za Zhi. (2006) 86:1011–3. doi: 10.3760/j:issn:0376-2491.2006.15.002 16784700

[145] Guo L Yu S Feng J. Analysis and optimization of development model of community sports public service under the perspective of active health. J Shijiazh Univ. (2019) 21:102–6. doi: 10.13573/j.cnki.sjzxyxb.2019.03.016

[146] Pavel M Jimison HB Korhonen I Gordon CM Saranummi N. Behavioral informatics and computational modeling in support of proactive health management and care. IEEE Trans Biomed Eng. (2015) 62:2763–75. doi: 10.1109/TBME.2015.2484286, PMID: 26441408 PMC4809752

[147] San Lucas N. Rojas D. (2014) A systematic review of proactive health management and empowerment for senior citizens. IEEE Long Island Systems, Applications and Technology (LISAT) Conference 2014

[148] Xiang-chen L Meng-sun Y. Proactive health: from idea to model. China Sport Sci. (2020) 40:83–9. doi: 10.16469/j.css.202002009

[149] Ye T Zhao Y Wang X Ling Y Wang H. Research on community chronic disease management model based on the concept of “active health”. Health Econ Res. (2021) 38:45–8. doi: 10.14055/j.cnki.33-1056/f.2021.08.011

[150] Zhang Y Li J Hu Y Liang S Wang Y Chen L. Proactive health behavior in middle-aged and older adult females with urinary incontinence: a grounded theory study. Neurourol Urodyn. (2024) 43:2005–16. doi: 10.1002/nau.25526, PMID: 38860474

[151] Du X Bao Y. The community health management development strategy in the situation of the new medical reform. Chin J Gen Pract. (2010) 8:1207–80. doi: 10.16766/j.cnki.issn.1674-4152.2010.10.004

[152] Liu B Zhang L. The present situation and influencing factors of continuous service in PHC. Chin Health Econ. (2006) 27:12–5. https://kns.cnki.net/kcms2/article/abstract?v=sfGpRh49pdFh2D0U1GpFm8HRk4UqL2lBtSulNgd1xr9h95mN7OCv88XAK7ZhIdkZqXjb2JbquIAOlaHNoMkU7rpvovVD02GOKDV5Q7w4BU6S4jt3JZCu3w5kqxNTtwmNjh0pHU91B7k2OFk1lg6ZWpcb6YWMgEmktzF7Wyw02iMBgAkjm1VnHw==&uniplatform=NZKPT&language=CHS

[153] Jiang Y. Impact of community proactive health management application on electronic health literacy and self-Management of Hypertensive Patients. Public Health Nurs. (2024) 41:1436–45. doi: 10.1111/phn.13424, PMID: 39297673

[154] Watt RG. From victim blaming to upstream action: tackling the social determinants of oral health inequalities. Community Dent Oral Epidemiol. (2007) 35:1–11. doi: 10.1111/j.1600-0528.2007.00348.x, PMID: 17244132

[155] Whitehead M. Dahlgren G. (2021). Concepts and principles for tackling social inequities in health: Levelling up Part 1. Copenhagen, Denmark: World Health Organization. Available online at: https://www.euro.who.int/en/publications/abstracts/concepts-and-principles-for-tackling-social-inequities-in-health-levelling-up-part-1

[156] Qian H. Analysis of the current situation and trends of the development of big health industry. Int J Biol Life Sci. (2023) 2:69–72. doi: 10.54097/ijbls.v2i3.8656

[157] Industry, Q. I. o. Q. (2024) China's strategic emerging industries in 2024- a panoramic atlas of the big health industry (with industrial scale, regional distribution, enterprise layout and cutting-edge technology, etc.). Available online at: https://bg.qianzhan.com/trends/detail/506/240524-cba718dc.html

[158] Kumar A Yadav JP Maheshwari S Singh A Srivastava V Khalilullah H. Revolutionizing healthcare with 5 G and AI: integrating emerging technologies for personalized care and cancer management. Intell Hospital. (2025):100005. doi: 10.1016/j.inhs.2025.100005

[159] Babarinde AO Ayo-Farai O Maduka CP Okongwu CC Ogundairo O Sodamade O. Review of AI applications in healthcare: comparative insights from the USA and Africa. Int Med Sci Res J. (2023) 3:92–107. doi: 10.51594/imsrj.v3i3.641

[160] Topol EJ. High-performance medicine: the convergence of human and artificial intelligence. Nature Medicine. (2019) 25:44–56. doi: 10.1038/s41591-018-0300-7 30617339

[161] Nawrat Z. Introduction to AI-driven surgical robots. AI Surg. (2023) 3:90–7. doi: 10.20517/ais.2023.14

[162] Syed Sarosh M Battineni G Khawaja M Allana R Maria KS a DA. How does artificial intelligence impact digital healthcare initiatives? A review of AI applications in dental healthcare. Int J Inform Manag Data Insights. (2023) 3:100144. doi: 10.1016/j.jjimei.2022.100144

[163] Amiri Z Taghavirashidizadeh A Parsa K. Ai-driven decision-making in healthcare information systems: a comprehensive review. J Syst Softw. (2025):112470. doi: 10.1016/j.jss.2025.112470

[164] Bao Y Gong W Yang K. A literature review of human–AI synergy in decision making: from the perspective of affordance actualization theory. Systems. (2023) 11:442. doi: 10.3390/systems11090442

[165] Sandeep Ganesh G Kolusu AS Prasad K Samudrala PK Nemmani KVS. Advancing health care via artificial intelligence: from concept to clinic. Eur J Pharmacol. (2022) 934:175320. doi: 10.1016/j.ejphar.2022.175320, PMID: 36220360

[166] Thakur A Mishra AP Panda B Rodríguez DCS Gaurav I Majhi B. Application of artificial intelligence in pharmaceutical and biomedical studies. Curr Pharm Des. (2020) 26:3569–78. doi: 10.2174/1381612826666200515131245, PMID: 32410553

[167] Reichenpfader D Rösslhuemer P Denecke K. Large language model-based evaluation of medical question answering systems: algorithm development and case study. Stud Health Technol Inform. (2024) 313:22–7. doi: 10.3233/shti240006 38682499

[168] Reicher L Lutsker G Michaan N Grisaru D Laskov I. Exploring the role of artificial intelligence, large language models: comparing patient-focused information and clinical decision support capabilities to the gynecologic oncology guidelines. Int J Gynaecol Obstet. (2025) 168:419–27. doi: 10.1002/ijgo.15869, PMID: 39161265 PMC11726133

[169] Yang H Li M Zhou H Xiao Y Fang Q Zhou S. Large language model synergy for ensemble learning in medical question answering: design and evaluation study. J Med Internet Res. (2025) 27:e70080. doi: 10.2196/70080, PMID: 40658884 PMC12337233

[170] Kale M Wankhede N Pawar R Ballal S Kumawat R Goswami M. AI-driven innovations in Alzheimer's disease: integrating early diagnosis, personalized treatment, and prognostic modelling. Ageing Res Rev. (2024) 101:102497. doi: 10.1016/j.arr.2024.102497, PMID: 39293530

[171] Chen Z Zhang D Liu C Wang H Jin X Yang F. Traditional Chinese medicine diagnostic prediction model for holistic syndrome differentiation based on deep learning. Integr Med Res. (2024) 13:101019. doi: 10.1016/j.imr.2023.101019, PMID: 38298865 PMC10826311

[172] Li D Qu J Tian Z Mou Z Zhang L Zhang X. Knowledge-based recurrent neural network for TCM cerebral palsy diagnosis. Evid Based Complement Alternat Med. (2022) 2022:1–10. doi: 10.1155/2022/7708376, PMID: 36276852 PMC9581687

[173] Wang L Tang K Wang Y Zhang P Li S. Advancements in artificial intelligence-driven diagnostic models for traditional Chinese medicine. Am J Chin Med. (2025) 53:647–73. doi: 10.1142/s0192415x25500259, PMID: 40374369

[174] Nguyen CV Li Y Bui TD Turner RE. Variational continual learning. arXiv. (2017) doi: 10.48550/arXiv.1710.10628

[175] Rebuffi S.-A. Kolesnikov A. Sperl G. Lampert C. H. (2017). ICARL: Incremental classifier and representation learning. Proceedings of the IEEE conference on Computer Vision and Pattern Recognition

[176] Rostami M Kolouri S Pilly P McClelland J. Generative continual concept learning. Proc AAAI Conf AI. (2020) 34:5545–52. doi: 10.1609/aaai.v34i04.6006

[177] Schwarz J. Czarnecki W. Luketina J. Grabska-Barwinska A. Teh Y. W. Pascanu R.. (2018). Progress & compress: A scalable framework for continual learning. International conference on machine learning. In Proceedings of the 35th International Conference on Machine Learning (Vol. 80, pp. 4528–4537). Stockholm, Sweden: PMLR. Available online at: https://proceedings.mlr.press/v80/schwarz18a.html

[178] Shin H. Lee J. K. Kim J. Kim J.. (2017). Continual learning through synaptic intelligence. International conference on machine learning. In Advances in Neural Information Processing Systems (Vol. 30, pp. 2990–2999). Red Hook, NY, USA: Curran Associates, Inc. Available online at: https://proceedings.neurips.cc/paper_files/paper/2017/file/0efbe98067c6c73dba1250d2beaa81f9-Paper.pdf

[179] Zenke F. Poole B. Ganguli S. (2017). Continual learning through synaptic intelligence. International conference on machine learning. In Proceedings of the 34th International Conference on Machine Learnings (Vol. 70, pp. 3980–3989). Sydney, Australia: PMLR Available online at: https://proceedings.mlr.press/v70/zenke17a.html PMC6944509 31909397

[180] Bae H. Song S. Park J. (2020). The Present and Future of Continual Learning. 2020 International Conference on Information and Communication Technology Convergence (ICTC), Jeju, Korea (South) 1193–1195. doi: 10.1109/ICTC49870.2020.9289549

[181] Iman M. Rasheed K. Arabnia H.R. (2021). 2021 International Conference on Computational Science and Computational Intelligence (CSCI) Las Vegas, NV, USA. pp. 190–192. doi: 10.1109/CSCI54926.2021.00103

[182] Kirkpatrick J Pascanu R Rabinowitz N Veness J Desjardins G Rusu AA. Overcoming catastrophic forgetting in neural networks. Proc Natl Acad Sci USA. (2017) 114:3521–6. doi: 10.1073/pnas.1611835114, PMID: 28292907 PMC5380101

[183] Lomonaco V. Maltoni D. Core50: a new dataset and benchmark for continuous object recognition. Conference on robot learning, (2017).

[184] Van de Ven GM Tolias AS. Three scenarios for continual learning. arXiv. (2019) doi: 10.48550/arXiv.1904.07734

[185] Liberati A Altman DG Tetzlaff J Mulrow C Gøtzsche PC Ioannidis JP. The PRISMA statement for reporting systematic reviews and meta-analyses of studies that evaluate health care interventions: explanation and elaboration. J Clin Epidemiol. (2009) 62:e1–e34. doi: 10.1016/j.jclinepi.2009.06.006 19631507

[186] Ritchie BW Jiang Y. Risk, crisis and disaster management in hospitality and tourism: a comparative review. Int J Contemp Hosp Manage. (2021) 33:3465–93. doi: 10.1108/IJCHM-12-2020-1480

[187] Rudnicka E Napierała P Podfigurna A Męczekalski B Smolarczyk R Monika G. The world health organization (WHO) approach to healthy ageing. Maturitas. (2020) 139:6–11. doi: 10.1016/j.maturitas.2020.05.018 32747042 PMC7250103

[188] Hamer M Lavoie KL Bacon SL. Taking up physical activity in later life and healthy ageing: the English longitudinal study of ageing. Br J Sports Med. (2014) 48:239–43. doi: 10.1136/bjsports-2013-092993, PMID: 24276781 PMC3913225

[189] Sadana R Blas E Budhwani S Koller T Paraje G. Healthy ageing: raising awareness of inequalities, determinants, and what could be done to improve health equity. Gerontologist. (2016) 56:S178–93. doi: 10.1093/geront/gnw034 26994259

[190] Prina CD. Physical activity and healthy ageing: a systematic review and meta-analysis of longitudinal cohort studies. Ageing Res Rev. (2017) 38:6–17. doi: 10.1016/j.arr.2017.06.003 28648951

[191] Szczechowicz B. The importance of attributes related to physical activity for the tourism product's utility. J Sport Tourism. (2012) 17:225–49. doi: 10.1080/14775085.2012.734061

[192] Giannake G Economou A Metaxas T Geitona M. Medical tourism in the region of Thessaly, Greece: opinions and perspectives from healthcare providers. Sustainability. (2023) 15:7864. doi: 10.3390/su15107864

[193] Lee LY-K Chu EC-P. Tai chi as a body-mind exercise for promotion of healthy aging in nursing home residents: appropriateness, feasibility, and effectiveness. Clin Interv Aging. (2023) 18:1949–59. doi: 10.2147/CIA.S430968 38020454 PMC10680471

[194] Ren Z He L Li X Yan L Ren Z Xiaolei L. Effect of traditional Chinese fitness exercises on bone mineral density of middle-aged and elderly people–a systematic review and network meta-analysis. Complement Ther Clin Pract. (2025) 59:101956. doi: 10.1016/j.ctcp.2025.101956 39908682

[195] Fuhong W Jinmei Z Hanxiao XU. Effects of new-style five-animal exercises on the balance ability and BMD of elderly women. Chinese Journal of Osteoporosis. (2018) Available online at: https://kns.cnki.net/kcms/detail/detail.aspx?dbcode=CJFD&dbname=CJFDLAST2019&filename=ZGGC201812017

[196] Shen Z-f Zhu G-f Qian L-f Fu Y-x. Yi jin jing (sinew-transforming qigong exercises) for primary osteoporosis in the elderly: a clinical trial. J Acupunct Tuina Sci. (2018) 16:104–8. doi: 10.1007/s11726-018-1032-4

[197] Song H. Effects of Taijiquan exercise on bone density and bone metabolism of primary osteoporosis sufferers. J Phys Educ. (2008) 11:106–8. doi: 10.16237/j.cnki.cn44-1404/g8.2008.11.027

[198] Sun B Chen R Ping Y Zhu Z Wu N He Y. Dynamic response of rock-like materials based on SHPB pulse waveform characteristics. Materials. (2022) 15:210. doi: 10.3390/ma15010210, PMID: 35009356 PMC8746182

[199] Wang Z Xu H Wang Z Wang Y Diao J Chen J. Traditional Chinese manual therapy (Tuina) improves knee osteoarthritis by regulating chondrocyte autophagy and apoptosis via the PI3K/AKT/mTOR pathway: an in vivo rat experiment and machine learning study. J Inflamm Res. (2024) 17:6501–19. doi: 10.2147/JIR.S488023, PMID: 39314229 PMC11417114

[200] Lee KE Choi M Jeoung B. Effectiveness of rehabilitation exercise in improving physical function of stroke patients: a systematic review. Int J Environ Res Public Health. (2022) 19:12739. doi: 10.3390/ijerph191912739, PMID: 36232038 PMC9566624

[201] Patti A Merlo L Ambrosetti M Sarto P. Exercise-based cardiac rehabilitation programs in heart failure patients. Heart Fail Clin. (2021) 17:263–71. doi: 10.1016/j.hfc.2021.01.007, PMID: 33673950

[202] Hoshino J. Renal rehabilitation: exercise intervention and nutritional support in Dialysis patients. Nutrients. (2021) 13:1444. doi: 10.3390/nu13051444, PMID: 33923264 PMC8145577

[203] Keteyian SJ Ehrman JK Fuller B Pack QR. Exercise testing and exercise rehabilitation for patients with atrial fibrillation. J Cardiopulm Rehabil Prev. (2019) 39:65–72. doi: 10.1097/hcr.0000000000000423, PMID: 30801433 PMC6394874

[204] Sanchez-Niubo A Forero C Wu Y Giné-Vázquez I Prina M De la Fuente J. Development of a common scale for measuring healthy ageing across the world: results from the ATHLOS consortium. Int J Epidemiol. (2021) 50:880–92. doi: 10.1093/ije/dyaa236 33274372 PMC8271194

[205] Kiefte-de Jong JC Mathers JC Franco OH. Nutrition and healthy ageing: the key ingredients. Proc Nutr Soc. (2014) 73:249–59. doi: 10.1017/S0029665113003881, PMID: 24503212

[206] Das G Kameswaran S Ramesh B Bangeppagari M Nath R Das Talukdar A. Anti-aging effect of traditional plant-based food: an overview. Foods. (2024) 13:3785. doi: 10.3390/foods13233785, PMID: 39682858 PMC11639806

[207] Li SY Lu ZH Leung J Su Y Yu B Kwok T. Dietary patterns modify the association between body mass index and mortality in older adults. Clin Nutr. (2025) 46:20–9. doi: 10.1016/j.clnu.2025.01.016, PMID: 39862690

[208] Giordano G Mastrantoni L Terranova R Colloca GF Zuccalà G Landi F. The role of Mediterranean diet in cancer incidence and mortality in the older adults. NPJ Aging. (2024) 10:61. doi: 10.1038/s41514-024-00186-w, PMID: 39639020 PMC11621705

[209] Veronese N Ragusa FS Dominguez LJ Cusumano C Barbagallo M. Mediterranean diet and osteoarthritis: an update. Aging Clin Exp Res. (2024) 36:231. doi: 10.1007/s40520-024-02883-8, PMID: 39625615 PMC11614952

[210] Ma Y Zhu Y Hong D Zhao H Li L. Association between tea drinking and disability levels in older Chinese adults: a longitudinal analysis. Front Nutr. (2023) 10:1233664. doi: 10.3389/fnut.2023.1233664, PMID: 38024372 PMC10644393

[211] Şimşek H Uçar A. Is ketogenic diet therapy a remedy for Alzheimer’s disease or mild cognitive impairments?: a narrative review of randomized controlled trials. Adv Gerontol. (2022) 12:200–8. doi: 10.1134/S2079057022020175

[212] Sakatani K Oyama K Hu L Warisawa S Yamashita T. Effects of exercise-diet therapy on cognitive function in healthy elderly people evaluated by deep learning based on basic blood test data. Adv Exp Med Biol. (2022) 1395:139–43. doi: 10.1007/978-3-031-14190-4_24, PMID: 36527628

[213] Donovan NJ Blazer D. Social isolation and loneliness in older adults: review and commentary of a national academies report. Am J Geriatr Psychiatry. (2020) 28:1233–44. doi: 10.1016/j.jagp.2020.08.005, PMID: 32919873 PMC7437541

[214] Fakoya OA McCorry NK Donnelly M. Loneliness and social isolation interventions for older adults: a scoping review of reviews. BMC Public Health. (2020) 20:129. doi: 10.1186/s12889-020-8251-6, PMID: 32054474 PMC7020371

[215] Stein Emil Vollset and Hazim. Burden of disease scenarios for 204 countries and territories, 2022–2050: a forecasting analysis for the global burden of disease study 2021. Lancet. (2024) 403:2204–56. doi: 10.1016/S0140-6736(24)00685-8 38762325 PMC11121021

[216] Holmes WR Joseph J. Social participation and healthy ageing: a neglected, significant protective factor for chronic non communicable conditions. Glob Health. (2011) 7:43. doi: 10.1186/1744-8603-7-43, PMID: 22035190 PMC3216238

[217] Bian S Tian X Meng F Xu C Zhao Y Gao Q. Assessing cognitive impairment in home-dwelling Chinese elders aged 80+: a detailed survey of 13,000 participants focusing on demographic factors, social engagement, and disease prevalence. Front Psych. (2024) 15:1355708. doi: 10.3389/fpsyt.2024.1355708, PMID: 38628263 PMC11019016

[218] Yang Q Lin S Zhang Z Du S Zhou D. Relationship between social activities and cognitive impairment in Chinese older adults: the mediating effect of depressive symptoms. Front Public Health. (2024) 12:1506484. doi: 10.3389/fpubh.2024.1506484, PMID: 39926291 PMC11802437

[219] Feng K Altinay L Zaid A. Social connectedness and well-being of elderly customers: do employee-to-customer interactions matter? J Hosp Market Manag. (2023) 32:174–95. doi: 10.1080/19368623.2023.2139036

[220] Liu W Xu W Wu M. The effect of tourist-to-tourist interaction on life satisfaction: a mediation role of social connectedness. Sustainability. (2022) 14:16257. doi: 10.3390/su142316257

[221] Yin Q Chen L Mao X Eva K. Restricted yet expanded: a case study of mobility adaptations among older adults with mild cognitive impairment in Zhengzhou, China. J Transp Health. (2025) 41:101992. doi: 10.1016/j.jth.2025.101992

[222] Yoo SY Han JM Park JH. Effect of social interaction intervention on the elderly: a systematic review. Alzheimers Dement. (2024) 20:e088015. doi: 10.1002/alz.088015

[223] Chan SHW Cheston R Steward-Anderson C Yu C-H Dodd E Coulthard E. Mindfulness meditation for sleep disturbances among individuals with cognitive impairment: a scoping review. Healthcare. (2025) 13:296. doi: 10.3390/healthcare13030296, PMID: 39942485 PMC11817335

[224] Haudry S Turpin AL Landeau B Mézenge F Delarue M Hébert O. Decoding meditation mechanisms underlying brain preservation and psycho-affective health in older expert meditators and older meditation-naive participants. Sci Rep. (2024) 14:29521. doi: 10.1038/s41598-024-79687-3, PMID: 39604423 PMC11603193

[225] Hung HY Azman A Jamir Singh PS. The impact of counseling on the dignity of older people: protocol for a mixed methods study. JMIR Res Protoc. (2023) 12:e45557. doi: 10.2196/45557, PMID: 37272062 PMC10337390

[226] Gross JJ Carstensen LL Pasupathi M Tsai J Götestam Skorpen C Hsu AY. Emotion and aging: experience, expression, and control. Psychol Aging. (1997) 12:590–9. doi: 10.1037//0882-7974.12.4.590, PMID: 9416628

[227] Grossi G Lanzarotti R Napoletano P Noceti N Odone F. Positive technology for elderly well-being: a review. Pattern Recogn Lett. (2020) 137:61–70. doi: 10.1016/j.patrec.2019.03.016

[228] Fredrickson LB Levenson RW. Positive emotions speed recovery from the cardiovascular sequelae of negative emotions. Cognit Emot. (1998) 12:191–220. doi: 10.1080/026999398379718, PMID: 21852890 PMC3156608

[229] Fredrickson BL. The role of positive emotions in positive psychology: the broaden-and-build theory of positive emotions. Am Psychol. (2001) 56:218–26. doi: 10.1037/0003-066X.56.3.218, PMID: 11315248 PMC3122271

[230] Zhao Y Liu Y Wang Z. Effectiveness of horticultural therapy in people with dementia: a quantitative systematic review. J Clin Nurs. (2022) 31:1983–97. doi: 10.1111/jocn.15204, PMID: 32017241

[231] Liu Z Ying W Yao C Ying F. Effects of aromatherapy on stress and emotion in office workers: an electroencephalogram analysis. DYNA. (2025) 100:56–62. doi: 10.52152/D8041, PMID: 38375865

[232] Jodie F Anna K Ursula W. Effectiveness of music therapy, aromatherapy, and massage therapy on patients in palliative care with end-of-life needs: a systematic review. J Pain Symptom Manag. (2025) 69:102–13. doi: 10.1016/j.jpainsymman.2024.07.024, PMID: 39142496

[233] Ben Massoued R Aissa A Larnaout A Lansari R Ouali U Jomli R. A case report of cognitive behavioural and emotional therapy for depression in an ultra-high risk patient. Eur Psychiatry. (2023) 66:S483–3. doi: 10.1192/j.eurpsy.2023.1033

[234] Paschos K. The management of chronic diseases is a major challenge for modern health systems: an integration of concepts and strategies concerning patients, doctors and health care. Sci Chron. (2020) 25:38–53. Available online at: http://www.tzaneio.gr/wp-content/uploads/epistimonika_xronika/p20-1-3.pdf

[235] Röhrich C Giordano J Kohls NB. Narrative view of the role of health promotion and salutogenesis in the treatment of chronic disease: viability and value for the care of cardiovascular conditions. Cardiovasc Diagn Ther. (2021) 11:591–601. doi: 10.21037/cdt-20-610, PMID: 33968636 PMC8102269

[236] Lee CW Li C. The process of constructing a health tourism destination index. Int J Environ Res Public Health. (2019) 16:4579. doi: 10.3390/ijerph16224579, PMID: 31752340 PMC6888581

[237] Crooks V Cameron K Chouinard V Johnston R Snyder J Casey V. Use of medical tourism for hip and knee surgery in osteoarthritis: a qualitative examination of distinctive attitudinal characteristics among Canadian patients. BMC Health Serv Res. (2012) 12:417. doi: 10.1186/1472-6963-12-417 23170924 PMC3515802

[238] Ali KF Mikhael A Zayouna C Barakat OA Bena J Lansang MC. Medical tourism and diabetes care: experience from a tertiary referral center. Endocr Pract. (2020) 26:1125–30. doi: 10.4158/EP-2020-0054, PMID: 33471714

[239] Liu S Wu S Qi J Wang L. Effect of traditional Chinese fitness exercises on bone mineral density in postmenopausal women: a network meta-analysis of randomized controlled trials. Front Endocrinol. (2024) 15:1323595. doi: 10.3389/fendo.2024.1323595, PMID: 38390196 PMC10882717

[240] Marshall AC. Traditional Chinese medicine and clinical pharmacology In: Drug discovery and evaluation methods in clinical pharmacology. Cham: Springer (2020). 455–482. doi: 10.1007/978-3-319-68864-0_60

[241] Zhu J Du X. Immune changes in “hepatitis inflammation and Cancer transformation process” from the perspective of traditional Chinese medicine. J Contemp Med Pract. (2024) 6:35–40. doi: 10.53469/jcmp.2024.06(07).08

[242] Li H-F Shen Q-H Chen W-J Chen W-M Feng Z-F Yu L-Y. Efficacy of traditional Chinese medicine tonifying kidney (Bushen) and activating blood (Huoxue) prescription for premature ovarian insufficiency: a systematic review and meta-analysis. Evid Based Complement Alternat Med. (2020) 2020:1789304. doi: 10.1155/2020/1789304, PMID: 32382277 PMC7191427

[243] Zhang Z. Traditional Chinese medicine: History, philosophy and treatment. J Tradit Complement Med. (2020) 10:113–118. doi: 10.1016/j.jtcme.2019.08.001

[244] Wu LL Wei C Zhao CC. “Discussion on qi and qi-collateral theory,” in Proceedings of the 17th International Congress on Luo Disease (pp. 36–42.). Chinese Association of Traditional Chinese Medicine. (2021). doi: 10.26914/c.cnkihy.2021.071858

[245] Singh K Gupta JK Jain D Kumar S Singh T Sunam S. Exploring the ancient wisdom and modern relevance of Chinese medicine: a comprehensive review. Pharmacol Res Modern Chin Med. (2024) 11:100448. doi: 10.1016/j.prmcm.2024.1004482024

[246] Pandey M Rastogi S Rawat A. Indian traditional ayurvedic system of medicine and nutritional supplementation. Evid Based Complement Alternat Med. (2013) 2013:376327. doi: 10.1155/2013/376327 23864888 PMC3705899

[247] Deng X Zhu H Shi L Li Y Shi H Wu Y. Comparison of the efficacy of acupuncture with tuina with acupuncture-only in the treatment of peripheral facial paralysis: a network meta-analysis. Intern Emerg Med. (2024) 19:839–58. doi: 10.1007/s11739-024-03562-2, PMID: 38483737 PMC11039505

[248] Rizvi SA Einstein GP Tulp OL Sainvil F Branly R. Introduction to traditional medicine and their role in prevention and treatment of emerging and re-emerging diseases. Biomolecules. (2022) 12:1442. doi: 10.3390/biom12101442, PMID: 36291651 PMC9599697

[249] Zhao X Tan X Shi H Xia D. Nutrition and traditional Chinese medicine (TCM): a system’s theoretical perspective. Eur J Clin Nutr. (2021) 75:267–73. doi: 10.1038/s41430-020-00737-w, PMID: 32884122

[250] Dawes NC Anastasi JK. The case for moxibustion for painful syndromes: history, principles and rationale. Curr Res Complement Alt Med. (2022) 6:153. doi: 10.29011/2577-2201.100053, PMID: 36147245 PMC9491495

[251] Mehta P Vividha D. Cupping therapy: a prudent remedy for a plethora of medical ailments. J Tradit Complement Med. (2015) 5:127–34. doi: 10.1016/j.jtcme.2014.11.036 26151023 PMC4488563

[252] Chen X Cui J Li R Norton R Park J Kong J. Dao yin (aka qigong): origin, development, potential mechanisms, and clinical applications. Evid Based Complement Alternat Med. (2019) 2019:3705120. doi: 10.1155/2019/3705120 31772593 PMC6854271

[253] Gómez-Lozano S Vargas-Macías A Kelly-Lahon C León K García-Sottile ME. Influence of aikido and Taijiquan-Tuishou on contact improvisation. Front Commun. (2022) 7:983290. doi: 10.3389/fcomm.2022.983290

[254] Ge RL Wood H Yang HH Liu YN Wang XJ Babb T. The body weight loss during acute exposure to high-altitude hypoxia in sea level residents. Sheng Li Xue Bao. (2010) 62:541–6. 21170501

[255] Burtscher M Gatterer H Burtscher J Mairbäurl H. Extreme terrestrial environments: life in thermal stress and hypoxia. A narrative review. Front Physiol. (2018) 9:572. doi: 10.3389/fphys.2018.00572, PMID: 29867589 PMC5964295

[256] Ellis A. Personality theories: critical perspectives In: Abrams M, editor. Thousand Oaks, CA, USA: Personal Theor Crit Perspect (2009)

[257] Xu Q Bauer R Hendry BM Fan T-P Zhao Z Duez P. The quest for modernisation of traditional Chinese medicine. BMC Complement Altern Med. (2013) 13:132. doi: 10.1186/1472-6882-13-132, PMID: 23763836 PMC3689083

[258] World Health Organization. WHO global report on traditional and complementary medicine. Geneva, Switzerland: World Health Organization (2019).

[259] Schnyer RN Allen JJ. Bridging the gap in complementary and alternative medicine research: manualization as a means of promoting standardization and flexibility of treatment in clinical trials of acupuncture. J Altern Complement Med. (2002) 8:623–34. doi: 10.1089/107555302320825147, PMID: 12470444

[260] Song P Xia J Rezeng C Tong L Tang W. Traditional, complementary, and alternative medicine: focusing on research into traditional Tibetan medicine in China. Bio Science Trends. (2016) 10:163–70. doi: 10.5582/bst.2016.01105, PMID: 27301588

[261] Li N Guo Y Gong Y Zhang Y Fan W Yao K. The anti-inflammatory actions and mechanisms of acupuncture from acupoint to target organs via neuro-immune regulation. J Inflamm Res. (2021) 14:7191–224. doi: 10.2147/JIR.S341581, PMID: 34992414 PMC8710088

[262] Yuan L Mingxiao Y Fan W Ke C Haiyong C Xueyong S. Mechanism of electroacupuncture on inflammatory pain: neural-im mune-endocrine interactions. J. Tradit. Chin. Med. (2019) 39:740–9. doi: 10.1016/S0254-6272(19)30084-8 32186125

[263] Pikkel Y Eliad H Ofir H Zeidan M Eldor L Nakhleh H. Mending a world of problems: 12-year review of medical tourism inbound complications in a tertiary Centre. Aesthet Plast Surg. (2025) 49:2492–7. doi: 10.1007/s00266-024-04523-y, PMID: 39870928

[264] Kaya S Sezerel H Filimonau V. How mindfulness training changes tourist experience: an exploratory study. J Hosp Tour Manag. (2024) 59:166–79. doi: 10.1016/j.jhtm.2024.04.007

[265] Nautiyal R Albrecht J Carr A. Spiritual practice as tourism experience: an application of cultural transmission theory. Ann Tourism Res. (2025) 110:103866. doi: 10.1016/j.annals.2024.103866

[266] Wang T Tang T Tsai C. The visual attention and psychological responses from older customers to wellness service pictures of hotels. Int J Environ Res Public Health. (2022) 19:1084. doi: 10.3390/ijerph19031084 35162108 PMC8834234

[267] McAuliffe PB Muss TEL Desai AA Talwar AA Broach RB Fischer JP. Complications of aesthetic surgical tourism treated in the USA: a systematic review. Aesth Plast Surg. (2023) 47:455–64. doi: 10.1007/s00266-022-03041-z, PMID: 36315261 PMC9619012

[268] Miyashita Y Akaleephan C Asgari-Jirhandeh N Sungyuth C. Cross-border movement of older patients: a descriptive study on health service use of Japanese retirees in Thailand. Glob Health. (2017) 13:14. doi: 10.1186/s12992-017-0241-9, PMID: 28274263 PMC5343304

[269] Lara J Cooper R Nissan J Ginty AT Khaw K-T Deary IJ. A proposed panel of biomarkers of healthy ageing. BMC Med. (2015) 13:222. doi: 10.1186/s12916-015-0470-9 26373927 PMC4572626

